# Merging the Energy Decomposition Analysis with the Interacting Quantum Atoms Approach

**DOI:** 10.1021/acs.jctc.3c00143

**Published:** 2023-05-29

**Authors:** Martí Gimferrer, Sergi Danés, Diego M. Andrada, Pedro Salvador

**Affiliations:** † Institut de Química Computacional i Catàlisi i Departament de Química, Universitat de Girona, c/ Maria Aurèlia Capmany i Farnés 69, 17003 Girona, Catalonia, Spain; ‡ Faculty of Natural Sciences and Technology, Department of Chemistry, Saarland University, 66123 Saarbrücken, Federal Republic of Germany

## Abstract

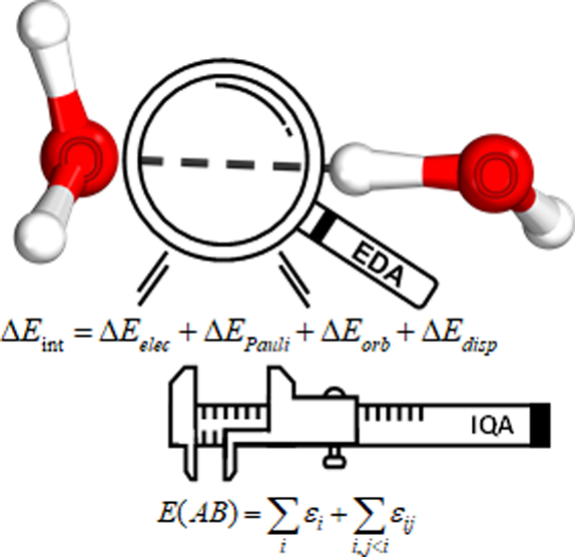

Energy decomposition analysis (EDA) is a well-established approach to dissect the interaction energy into chemically sound components. Despite the inherent requirement of reference states has been a long-standing object of debate, the direct relation with the molecular orbital analysis helps in building up predictive models. The alternative molecular energy decomposition schemes that decompose the total energy into atomic and diatomic contributions, such as the interacting quantum atoms (IQA), has no external reference requirements and also the intra- and intermolecular interactions are treated on equal footing. However, a connection with heuristic chemical models are limited, bringing about a somewhat narrower predictive power. While efforts to reconcile the bonding picture obtained by both methodologies have been discussed in the past, a *synergic combination of them has not been tackled yet*. Herein, we present the use of IQA decomposition of the individual terms arising from the EDA in the context of intermolecular interactions, henceforth EDA–IQA. The method is applied to a molecular set covering a wide range of interaction types, including hydrogen bonding, charge–dipole, π–π and halogen interactions. We find that the electrostatic energy from EDA, entirely seen as intermolecular, leads to meaningful and non-negligible intra-fragment contributions upon IQA decomposition, originated from charge penetration. EDA–IQA also affords the decomposition of the Pauli repulsion term into intra- and inter-fragment contributions. The intra-fragment term is destabilizing, particularly for the moieties that are net acceptors of charge, while the inter-fragment Pauli term is actually stabilizing. In the case of the orbital interaction term, the sign and magnitude of the intra-fragment contribution at equilibrium geometries is largely driven by the amount of charge transfer, while the inter-fragment contribution is clearly stabilizing. EDA–IQA terms show a smooth behavior along the intermolecular dissociation path of selected systems. The new EDA–IQA methodology provides a richer energy decomposition scheme that aims at bridging the gap between the two main distinct real-space and Hilbert-space methodologies. Via this approach, the partitioning can be used directionally on all the EDA terms aiding in identifying the causal effects on geometries and/or reactivity.

## Introduction

Understanding and accurately assessing intra- and intermolecular interactions is one of the main challenges in chemistry. In fact, the rational design of molecular systems consists of unravelling the physical origin of a particular chemical interaction/bond, often inaccessible directly from experiments. However, a common drawback is the absence of an exact quantum-mechanical operator that directly describes the *chemical bond,* giving raise to different approaches.

Among the number of developments, some methods focus on the analysis of the electron density in a system (*AB*) by comparing it with that from the composing fragments (*A* and *B*). The concept of deformation density^1^ is commonly invoked in methods such as Voronoi deformation density charges^2^ or charge displacement analysis,^3,4^ for instance. The electron density of the *AB* system is a crucial component in the quantum theory of atoms in molecules^5^ (QTAIM). By analyzing its topology, QTAIM provides a plethora of descriptors that can be utilized to classify various intra- and intermolecular interactions.

A better option is to focus directly on the energetics of bond formation and intermolecular interactions. Modern electronic structure methods are able to predict accurate formation energies, but the value itself bears little chemical significance. Energy decomposition schemes aim at decomposing the molecular (or formation) energy into physicochemical meaningful terms, to shed light into the nature of the chemical bonding. By comprehending the individual contributions to the overall energy, it becomes possible to rationally design molecular systems with desired properties, leading to a more predictive approach in molecular design.

One of the most widely used methodologies is the Ziegler–Rauk energy decomposition analysis (EDA),^6^ derived from the pioneering work of Kitaura and Morokuma.^7^ It considers the formal molecule formation *AB* (henceforth complex) from fragments *A* and *B* (atoms or molecular fragments). The overall stabilization energy (without basis set superposition error correction) reads as
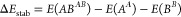
1where *E*(*X*^*Y*^) refers to the energy of the subsystem *X* at the optimized geometry of *Y*. Thus, Δ*E*_stab_ is the energy of formation of the system *AB* from the isolated fragments in their ground states *A* and *B*. Within the EDA formalism, Δ*E*_stab_ (eq 1) is decomposed as follows
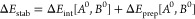
2being Δ*E*_int_ and Δ*E*_prep_ the so-called interaction and preparation energy terms, respectively, which are defined as
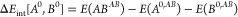
3
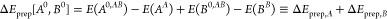
4Here, *E*(*A*^0,*AB*^) represents the energy of fragment *A* computed at the optimized geometry of the complex (superindex *AB*) with a given electronic configuration (*A*^0^), which may not correspond to that of the ground state for the isolated fragment. Defined as such, the preparation energy accounts for *both* the geometrical distortion of the fragments upon formation of the complex and the *promotion* energy from the electronic ground state to the chosen electronic configurations *A*^0^ and *B*^0^. One often refers to strain energy when it only involves the geometrical deformation. Furthermore, it is necessarily positive (repulsive or destabilizing) because *E*(*A*^*A*^) and *E*(*B*^*B*^) are, *by definition*, the ground state energies of the isolated fragments from both the electronic and geometric perspective. Instead, Δ*E*_int_ will be negative (attractive or stabilizing) if the interaction between the fragments *A* and *B* while forming complex *AB* is favorable. Importantly, both the interaction and preparation energies depend on the choice for the states *A*^0^ and *B*^0^ as indicated in eqs 3 and 4, being crucial its appropriate selection (see below for further details).

By introducing additional intermediate (pseudo)states built up at the optimized geometry of the complex, the interaction energy is further decomposed. Firstly, one considers a pseudostate of complex *AB* formed by the superposition of the undeformed (frozen) densities associated to the fragments in states *A*^0^ and *B*^0^, namely (*A*^0^ ∪ *B*^0^), with its associated electronic energy *E*(*A*^0^ ∪ *B*^0^). We refer to (*A*^0^ ∪ *B*^0^) as a pseudostate because it does not have a well-defined antisymmetric wavefunction associated to it.^6,8^ The energy difference with respect to the deformed fragments read as
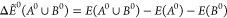
5which can be further expressed as
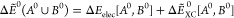
6where the *AB* superindex has been omitted for clarity. The term Δ*E*_elec_[*A*^0^, *B*^0^] accounts for the electrostatic interaction of the frozen electron density of fragment *A* with the nuclei of fragment *B* and vice versa (attractive), the Coulombic repulsion of the frozen electron densities of *A* and *B* and the nuclear repulsion between *A* and *B*.
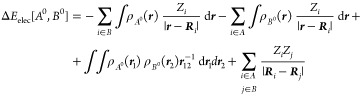
7

Equation 7 may be also rewritten as
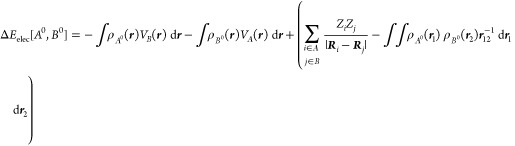
8where the molecular electrostatic potential (MEP) of fragment *A* in state *A*^0^, *V*_*A*_(*r*), reads as
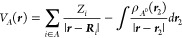
9

The potential originated from charge clouds is smaller than the one from point charges (nuclei) so that for neutral species the MEP of the fragments afford a favorable interaction that, at chemically relevant distances, overcomes the nuclear repulsion term.^9^ In Kohn–Sham density functional theory (KS-DFT) there is an additional contribution from the exchange–correlation functional,^8,9^ which is absent in wavefunction theory.

In a subsequent step, an intermediate state (*A*^0^*B*^0^) is formed by Löwdin orthogonalizing the occupied molecular orbitals (MOs) of the states (*A*^0^) and (*B*^0^), in order to build a proper antisymmetrized wavefunction. Orthogonalization is required as MOs belonging to different fragments are not orthogonal (in principle one could build a Slater determinant with non-orthogonal MOs but then the expectation value of the energy takes a much complicated form). The Löwdin orthogonalization procedure does not induce charge transfer between the fragments, as the Hilbert-space based electron numbers of the interacting fragments are conserved. This will not be the case when applying a real-space analysis, as will be discussed later.

The energy difference between this intermediate step and that of the previous pseudostate reads as
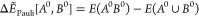
10which upon combination of eqs 6 and 10 leads to the so-called Pauli repulsion term (Δ*E*_Pauli_)^8^
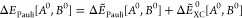
11

Hence, the sum Δ*E*_Pauli_[*A*^0^, *B*^0^] + Δ*E*_elec_[*A*^0^, *B*^0^] accounts for the energy change when going from the prepared fragments to the *true* intermediate state with orthogonalized but unrelaxed MOs, and it is a well-defined quantity in the sense that involves properly antisymmetrized states
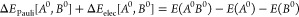
12

It is worth to note that  is never considered, as the  term is not evaluated explicitly. Once the electrostatic contribution is calculated using eq 7 or 8, the Pauli repulsion term is readily obtained from eq 12.

In the last step, the MOs of the complex are allowed to relax to the *ground state* of the complex. The energy lowering accompanying this process leads to the so-called orbital interaction term (Δ*E*_orb_), that is necessarily negative (any intermediate state must be higher in energy than the ground state)
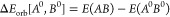
13

**Scheme 1:**
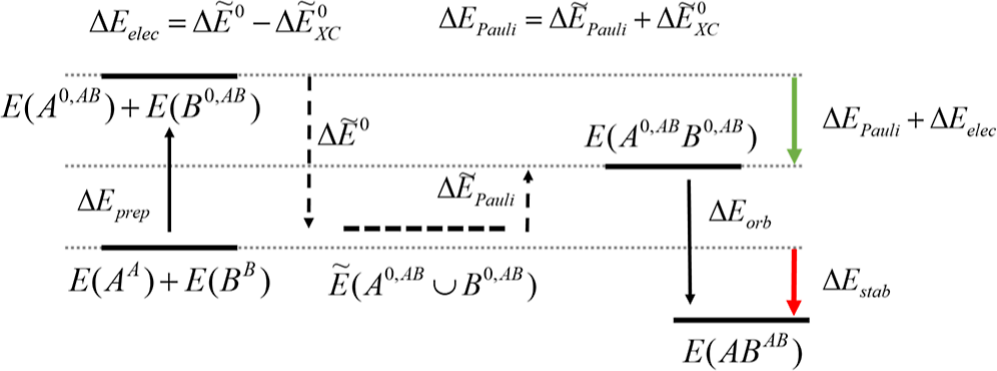
Energy Components of the EDA Process with the Corresponding Intermediate States and Pseudostates for the Formation of Complex *AB* from the Isolated Fragments *A* and *B* *E*(X^Y^) refers to the energy of subsystem *X* at the state and optimized geometry of *Y*.

All the steps along the EDA process are generally illustrated as in Scheme 1.

Localized orbitals can also be introduced in this type energy decomposition schemes. In the absolutely localized molecular orbitals EDA (ALMO-EDA),^10−13^ the interaction energy is further decomposed into a frozen-density, polarization and charge-transfer terms by making use of (variationally optimized) block-localized orbitals, and explicitly avoiding any reference to intermediate pseudostates. Natural EDA^14^ (NEDA) makes use of the well-known natural bond orbitals (NBO).^15−18^ In the symmetry adapted perturbation theory (SAPT) schemes the interaction energy is perturbationally computed, thus avoiding the supermolecular approach, and decomposed into physically meaningful terms.^19^ For further details about these EDA-like methodologies we guide the reader to refs (20) and (21).

Alternatively, the total energy of any molecular system can also be decomposed into intra- and inter-atomic contributions. Such decomposition does not require external references or predefined fragments, and treat intra- and inter-molecular interactions (covalent and non-covalent) on equal footing. Grouping the one- and two-center terms into intra- and inter-fragment contributions is only optional, but helpful in the case of dealing with intermolecular interactions between well-defined subsystems. The grouping of specific domains within the interacting fragments also allows the identification of main contributors and their mutual interactions. In Mayer’s Chemical Hamiltonian Approach, atomic projector operators are used to decompose the Hamiltonian into one- and two-center terms.^22^ Further developments in the Hilbert-space ultimately lead to the chemical energy component analysis.^23^ Considering instead a decomposition of the real-space, the one- and two-electron contributions to the total energy readily afford one- and two-center terms that exactly decompose (up to numerical accuracy) the molecular energy. Such methodologies rely on the identification of the atom within the molecule (AIM). Salvador and Mayer first decomposed the Hartree–Fock energy in the framework of QTAIM,^19^ paving the way for the nowadays known as the interacting quantum atoms approaches.^24−31^

In real-space analysis, a given quantity, *F*_1_, expressed in terms of a one-electron density function, *f*(***r***_1_), is readily decomposed into one-center (atomic or fragment) contributions
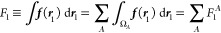
14by integrating over the respective domains. Similarly, two-electron quantities decompose into both one- and two-center components
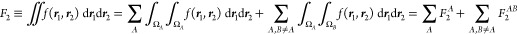
15

It is worth noting that real-space analysis is not restricted to non-overlapping disjoint domains such as those of QTAIM, where each atom is identified by its nucleus and its atomic basin. The AIM may be more generally represented by continuous atomic weight functions *w*_*A*_(**r**) ≥ 0 fulfilling Σ_Α_*w*_*A*_(***r***) = 1 so that the integration of molecular density functions over the atomic domains are effectively replaced by integrations over the whole real-space of atomic/diatomic effective density functions
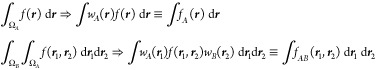
16

Such atomic weight functions can be derived from a variety of Hirshfeld-type approaches^32^ or even mathematical constructs borrowing elements of QTAIM theory.^33^ Whether the AIM are allowed to overlap or not might be to some extent matter of taste. Using one or another AIM only has an effect on the actual numerical values obtained for the terms obtained by the IQA decomposition, but not on their definition and physical meaning.

Since the Born–Oppenheimer energy is entirely written in terms of one- and two-electron energy density functions, IQA naturally affords the decomposition of the molecular energy of a complex *AB* into atomic and diatomic contributions as
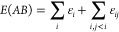
17where the ε_*i*_ and ε_*ij*_ terms account for the static net atomic and pairwise atomic interaction energies, respectively. The atomic and diatomic terms can be further grouped according to the composing fragments *A* and *B*, so that the total energy of the complex can be simply expressed as
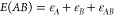
18where
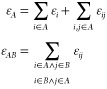
19and
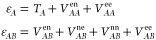
20

Each of the intra- and inter-fragment energy term is built upon the physical components of the electronic energy, i.e. kinetic, electron nuclear attraction, nuclear repulsion and electronic repulsion. The latter may be further decomposed into the usual Coulomb, exchange and correlation contributions. One peculiarity of the IQA-type approaches is that the actual formulation depends upon the particular electronic structure method that is used to compute the molecular energies in the first place. Appropriate formulations have been developed for Hartree–Fock^24,26,34^ and correlated methods (including CASSCF and CI,^27^ MP2^28,35^ and Coupled Cluster^29,30,36^). Non-perturbative approaches explicitly require the second order reduced density matrix, which is not available in most electronic structure codes. Curiously, the KS-DFT case is the most problematic one because of the exchange–correlation energy nature. Within wavefunction theory framework, the exchange energy is expressed as a two-electron non-local contribution, that naturally decomposes into both one- and two-center terms. The later are essential to account for the stability of the diatomic bonding interactions.^21^ In KS-DFT, the exchange–correlation energy is essentially written in terms of the exchange energy density as
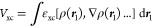
21so that the straightforward real-space decomposition only affords one-center (atomic) terms. Different approaches have been introduced to (approximately) recover the chemically meaningful diatomic components from *V*_xc_.^25,31^

Both EDA and IQA methodologies independently have been extensively used in the literature to gain deeper insight into the nature of the chemical bond and to characterize intra- and intermolecular interactions, allowing to understand and improve chemical reactivity, shedding light to the chemical-bonding picture of non-trivial systems and even most recently suggesting a new type of bond.^37−42^ Recent efforts have been made trying to express some of the EDA-derived descriptors in the framework of real-space analysis, i.e. without recurring to any artificial intermediate pseudostates. In this direction, in 2006 Martiín Pendás et al. compared the behavior of IQA to that of other decomposition schemes (e.g. EDA, NEDA and SAPT) for a series of hydrogen-bonded dimers.^43,44^ The authors decomposed the interaction energy between the two monomers *A* and *B* (*E*_int_^*AB*^) into the sum of classical electrostatics (*V*_cl_^*AB*^) and exchange–correlation (*V*_xc_^*AB*^) contributions. They observed that the interaction was governed by the exchange–correlation, thus highlighting the importance of the covalent picture. On the other hand, the deformation energy of the proton acceptor moieties correlated well with the intermolecular charge transfer and classical electrostatic energy derived from IQA. Furthermore, by making use of the fragment’s promolecular, polarized (by locating point charges) and fully relaxed densities, they observed that in weakly-bonded (almost non-overlapping) systems the quantities defined by other energy decomposition schemes, i.e. SAPT, KM, EDA and specially NEDA, can be obtained to a good approximation from the inter-fragment (*AB*) IQA terms. For instance, the electrostatic energy from NEDA was found to be roughly equivalent to the total inter-fragment interaction from IQA.^44^

Pendás et al. also critically analyzed the concept of steric repulsion from an IQA perspective.^45^ The authors argued that Pauli repulsion is inherently dependent on the fragment’s reference states in EDA. They applied IQA to decompose the Hartree–Fock interaction energy into fragment’s deformation and inter-fragment interactions
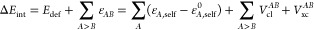
22where the latter is further decomposed into its classical electrostatic and exchange–(correlation) contributions. The authors concluded that the Pauli repulsion is readily captured in the increase of the fragment’s deformation energies of the intermediate (properly antisymmetrized) states. In the case of rotational barriers, the hyperconjugative effects are captured by the inter-fragment exchange contribution, enhanced due to electron delocalization. All in all, they show a certain degree of correspondence between EDA or NBO descriptors and those steaming from IQA.

More recently, Racioppi et al. walked a reverse path. Instead of recovering EDA descriptors from IQA, they rearranged the EDA contributions to match those of IQA analysis.^46^ In particular, in their pseudo-IQA energy decomposition the EDA contributions of Pauli repulsion, orbital interaction and electrostatic to the interaction energy are regrouped into overall variations of the kinetic, classical electrostatic and exchange–correlation contributions
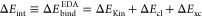
23

The same terms can be obtained by considering the usual reference-state IQA, which is based on decomposing the binding energy between two fragments *A* and *B* (Δ*E*_bind_^IQA^) by subtracting the IQA terms from the fully relaxed complex’s state from those obtained for the isolated fragments at the complex geometry. The authors showed excellent agreement between the like terms of both schemes in illustrative hydrogen bond and donor–acceptor interactions.^46^

In this work, we pursue a different path, namely to enrich the conventional EDA approach by applying an IQA decomposition to *each of the EDA terms* of the interaction energy. Thus, in the EDA-IQA scheme we introduce herein, the electrostatic, Pauli repulsion and orbital interaction energy terms are decomposed into intra- and inter-atomic contributions, that can be further grouped into intra- and inter-fragment contributions.

## Theory

Let us consider again the formation of the complex *AB* from fragments *A* and *B*. The application of eqs 14–16 to the complex’s final ground state (*AB*) readily affords the real-space decomposition of the interaction energy into intra- and inter-fragment terms, namely
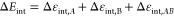
24where
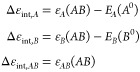
25

For clarity, in the previous equation and henceforth we omit the explicit dependence of the EDA term on the reference states (*A*^0^ and *B*^0^). The Δε_int,*A*_ and Δε_int,*B*_ account for the energy gain/loss by the fragments when going from their isolated reference state to their *effective* state within the final complex. It is worth to note that in the context of real-space analysis, these contributions do not only originate from changes in the MOs upon complex formation, but also by the fact that the fragments share the physical space once the complex is formed (in intermolecular interactions the second effect should be dominant). In ref (44) the authors refer to these terms as fragment’s electronic deformation energies. We will adopt here this nomenclature, so that Δε_int,*A*_ ≡ Δε_def.el,*A*_ and Δε_int,*B*_ ≡ Δε_def.el,*B*_.

On the other hand, the term Δε_int,*AB*_ describes the energy gain upon complex formation that can be purely ascribed to inter-fragment interactions. The net interaction energy is thus seen as a balance between the prize the fragments must pay to share the physical space and be electronically prepared, and the gain originating from the new interactions that were absent before the complex’s formation.

In a similar fashion, by applying again eqs 14–16 to the complex’s intermediate state (*A*^0^*B*^0^) one can also obtain an analogous decomposition of the orbital interaction EDA term, namely
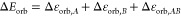
26where
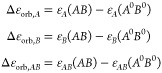
27

The intra-fragment terms account for the net energy gain/loss upon relaxing the wavefunction from the intermediate state to the ground state of the *AB* complex. This relaxation comes with a change in the electron density. If the underlying AIM definition depends upon this scalar (e.g. QTAIM, TFVC or iterative Hirshfeld approaches), these terms contain also a contribution from the change on the boundaries of physical space going from *AB* to *A*^0^*B*^0^. The latter could be removed by using the *same* AIM definition for states *AB* and *A*^0^*B*^0^. In the QTAIM context that means integrating the density functions of state *A*^0^*B*^0^ on the atomic basins obtained from the *AB* state. In the case of overlapping AIM schemes, it implies using the same atomic weight functions throughout. Such strategies have been already used in the context of QTAIM and fuzzy atoms in similar contexts.^44,47,48^ In the present case, since it is actually impossible by construction to use the same AIM definition for the complex and the isolated fragments, we opt for using the AIM definition derived from each state.

The IQA decomposition of state (*A*^0^*B*^0^) readily affords an analogous decomposition of Δ*E*_Pauli_ + Δ*E*_elec_, by taking the isolated fragment states *A*^0^ and *B*^0^ as reference. On the other hand, since each term in Δ*E*_elec_ involves the electron density and/or potential from different fragments (see eq 7), one may argue that this term is entirely of intermolecular nature. In that case, Δ*E*_elec_ would contribute solely to the inter-fragment term, and consequently one would have the following decomposition for Δ*E*_Pauli_
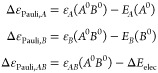
28

However, such scheme is not satisfactory neither numerically nor conceptually. The main concern is that Δε_Pauli,*AB*_ thus defined mixes up real-space and Hilbert-space quantities, while in this case they behave quite differently. Indeed, as mentioned above, there is no net charge-transfer between fragments *A* and *B* when building the intermediate state *A*^0^*B*^0^ according to Hilbert-space analysis (e.g. Mulliken and Löwdin populations add up to the number of electrons of each fragment). This is not the case when performing a real-space analysis (using disjoint or fuzzy domains), again because the fragments within the complex share the physical space
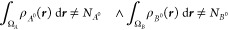
29

**Figure 1:**
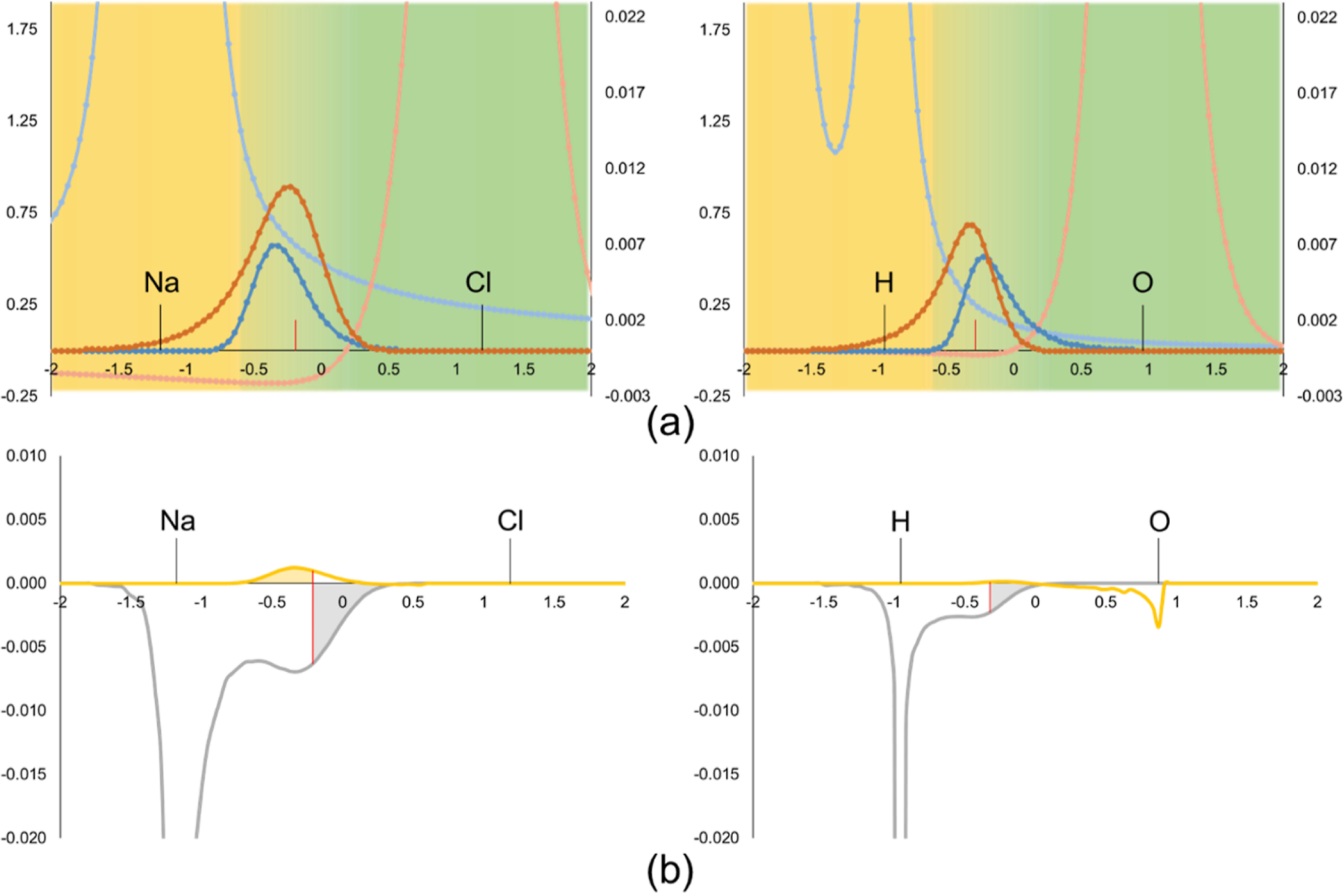
Potential and electron density profiles for NaCl and water dimer along the Na–Cl and the intermolecular H···O bonds, respectively. (a) Topology of  (light blue),  (light orange),  (dark blue) and  (dark orange). (b) Topology of  (grey) and  (yellow). Atomic (fuzzy) domains depicted as surface, yellow for *A* and green for *B*. Bond critical point depicted as red vertical line. Geometries and wavefunctions evaluated at the BP86-D3(BJ)/def2-TZVPP level of theory. Fragment definition: *A* = Na^+^, *B* = Cl^–^ (NaCl) and *A* = HO–H, *B* = OH_2_ (H_2_O···H_2_O).

Hence, the frozen density of isolated fragment *A* when brought to the complex’s geometry does not entirely *belong* to fragment *A*, and similarly for fragment *B*. This influences the numerical values obtained using eq 27 and, for consistency, this effect should be also taken into account when applying the real-space analysis to the other EDA terms, and in particular to Δ*E*_elec_. One should essentially ignore the original allegiance of the fragment’s frozen densities and treat the integrand in the exactly same manner as one does it with the electron-nuclear and the Coulombic contributions to the energy in the conventional IQA scheme, namely
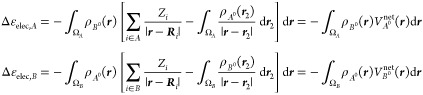
30Here, we introduce the fragment’s *net* electrostatic potentials *V*_*A*_^net^(***r***) and *V*_*B*_^net^(***r***). They are different from the electrostatic potentials *V*_*A*_(***r***) and *V*_*B*_(***r***) of eq 9, because in the electronic term the integration is carried out within the fragment’s domain. Since only part of the fragment density is used, *V*_*A*_^net^(***r***) > *V*_*A*_(***r***). Figure 1 depicts the topology of the net electrostatic potentials and fragment’s densities along the inter-fragment distance for NaCl and water dimer. In the vicinity of the nuclei, the electrostatic potential is large and positive, as the electronic part cannot compensate for the nuclear term. At longer distances from the nucleus, the net potential slowly decays to zero. However, in the case of a strong acceptor or an anionic fragment, since there is an excess of electron charge compared to the nuclear one, the net potential becomes *negative,* and tends to zero from below (see light orange curves in Figure 1). This effect is much more pronounced when the donor of charge is anionic (Cl^–^ vs H_2_O). On the other hand, there is a fraction of electron density of *B* that penetrates into *A* (i.e. *w*_*A*_(***r***)ρ_*B*^0^_(***r***)) and vice versa. It corresponds to the dark orange and dark blue curves in Figure 1. As expected, the density of the charge donor penetrates more and *deeper* into the acceptor region than the other way around. The interaction of that density from *B* with the net potential of the acceptor *A* results in the electrostatic contribution assigned to *A*, Δε_elec,*A*_. It corresponds to the integration of the grey curve in Figure 1b. This term is negative for the acceptor (notice the negative sign on the r.h.s. of eq 30) and can be significant if the density of the donor is able to penetrate deep into the acceptor’s domain. However, in the case of the donor, the net potential can be negative in the region where it interacts with the density penetrating from the acceptor *A*, so it might result in a (small) positive Δε_elec,*B*_ contribution, as shown by the yellow curve in Figure 1b in the case of Cl^–^.

In the case of the inter-fragment contribution one obtains
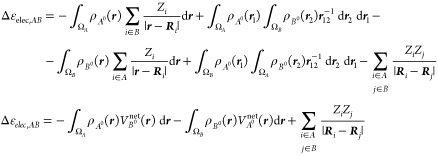
31

The numerical value of this contribution will account to which extent the net potential of fragment *A* penetrates into fragment *B* and vice versa, compensated by the point charge nuclear repulsions. As we will see, this term can be positive of negative. In any case, one can readily see that
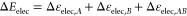
32

It is fair to note that Jiménez-Grávalos and Suárez recently achieved a similar decomposition of the electrostatic interaction in the QTAIM framework for a different purpose.^49^ They did not explicitly considered fragment’s electrostatic potentials, but it can be seen that their *E*_ele_^*A*^(ρ_*A*_^0^, ρ_*B*_^0^) and *E*_ele_^*B*^(ρ_*A*_^0^, ρ_*B*_^0^) terms correspond to our Δε_elec,*A*_ and Δε_elec,*B*_, respectively. Jiménez-Grávalos and Suárez further decompose the inter-fragment electrostatic contribution into a dominant term *E*_ele_^*AB*^(ρ_*A*_^0^, ρ_*B*_^0^) that tends to the overall Δ*E*_elec_ at long distances, and a residual one *E*_ele_^*BA*^(ρ_*A*_^0^, ρ_*B*_^0^) which, together with the intra-fragment contributions, accounts for the charge-penetration energy. We shall see that Δε_elec,*AB*_ from eq 31 also converges smoothly to Δ*E*_elec_ at long inter-fragment distances, so for the present purpose we do not consider such additional decomposition.

Subtracting the contributions of eq 32 from those originating from the IQA decomposition of Δ*E*_Pauli_ + Δ*E*_elec_ finally yield the appropriate real-space decomposition of the Pauli repulsion term
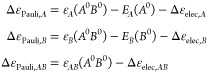
33

Fulfilling again the sum rule
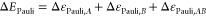
34

The final EDA picture is completed by the inclusion of the preparation energies from eq 4 and, if required, a dispersion correction. In the case of the semiempirical dipole–dipole model of Grimme, the dispersion correction is added to the interaction energy and has no influence in the intermediate steps, being trivially decomposed by construction as
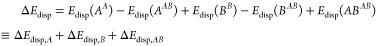
35

This will not be the case if one uses more sophisticated density-dependent dispersion corrections such as VV10.^50^ Finally, the basis set superposition error (BSSE) correction can be estimated *a posteriori* via the counterpoise formula,^51,52^ which is also additive
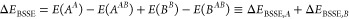
36

Summarizing, the present approach affords a real-space fully additive decomposition into intra- (*A* or *B*) and inter-fragment (*AB*) contributions of all terms occurring in the EDA scheme
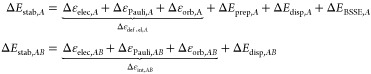
37

## Computational Details

All KS-DFT calculations were performed with the Gaussian16 A03 software,^53^ using the gradient corrected BP86 functional from Becke and Perdew,^54,55^ including dispersion correction to the electronic energy by means of Grimme D3^56^ with Becke–Johnson (BJ) damping function,^57^ and the Ahlrichs def2-TZVPP full electron basis set.^58^ Geometry optimizations were performed without symmetry constrains for all systems. Stationary points were characterized by computing analytical Hessians, obtaining zero imaginary frequencies in all cases (minima).

To construct all EDA states, the wavefunctions of the dimer and the isolated fragments at the optimized and dimer geometries were evaluated with Gaussian16. The pseudostate (*A*^0^ ∪ *B*^0^) electronic structure was constructed using the MOs of the isolated fragments at the dimer geometry. This step was performed with the local program APOST-3D,^59^ providing its electronic structure information in a formatted checkpoint (.fchk). Transformation of the formatted into unformatted (.chk) checkpoint file was realized with the *unfchk* tool from Gaussian. Finally, its associated total energy was extracted using the created chk file as starting guess and forcing to skip the SCF procedure (e.g. SCF = (MAXCYCLE = −1) keyword in Gaussian16). In these calculations the symmetry use was fully disabled to prevent any atomic basis set position difference.

Energy decomposition calculations were also performed with the APOST-3D code using the TFVC atomic definition.^33^ For the production results, one-electron numerical integrals were realized using 150 radial (Gauss–Legendre quadrature^60^) and 974 angular Lebedev–Laikov^61^ grid points per atom, while two-electron numerical integrals have been performed using 150 radial and 590 angular grid mesh. Numerical error minimization of the one-center two-electron contributions was performed using the zero-error strategy.^62^

## Results and Discussion

We have considered the set of intermolecular complexes from ref (20), that essentially includes hydrogen bonded species, cation-dipole, cation−π, halogen−π and π–π interactions between dimers. In addition, we have also considered several anion-π complexes from Quiñonero et al.^63^ Except for the π–π interactions, one can identify electron donor and acceptor moieties, which entails certain charge-transfer upon complex formation. We will henceforth refer to fragment *A* as the net acceptor of charge and fragment *B* as the donor of charge.

Let us start by analyzing the electrostatic contribution of EDA. Table 1 gathers the IQA decomposition of the electrostatic contributions for the whole set of systems at equilibrium. Note how cation−π and anion−π interactions result in similar values of Δ*E*_elec_, but their IQA decomposition reveals a completely different mechanism. In the former, the cation is largely stabilized (large and negative Δε_elec,*A*_ values) upon complex formation because of its interaction with the frozen density of the donor. This is largely compensated by a positive inter-fragment electrostatic term Δε_elec,*AB*_, that becomes more repulsive as the equilibrium inter-fragment distance shortens from K^+^ to Li^+^. In the case of anion−π interactions, the Δε_elec,*B*_ contribution is positive, in line with the situation of Cl^–^ in NaCl previously depicted in Figure 1, and the inter-fragment term is positive. In dispersion bound systems, both the overall electrostatic and their IQA components are very small, and in most cases within numerical accuracy. In the hydrogen bonded and cation-dipole systems, one cannot see a clear trend neither for the intra-fragment contributions (in almost all cases negative) nor for the Δε_elec,*AB*_ values.

**Table 1:** Fragment (IQA) Decomposition of the Δ*E*_elec_ Term From EDA of the Systems Studied^a^

A = acceptor	B = donor	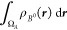	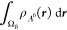	Δε_elec,*A*_	Δε_elec,*B*_	Δε_elec,*AB*_	Δ*E*_elec_
H_2_O	H_2_O	0.050	0.043	–6.1	–0.6	–2.2	–8.9
H_2_O	MeOH	0.055	0.050	–6.8	–0.9	–1.6	–9.4
MeOH	MeOH	0.058	0.052	–7.2	–1.1	–1.3	–9.7
H_2_O	NH_3_	0.080	0.045	–10.2	–0.4	–2.3	–12.9
NH_4_^+^	H_2_O	0.077	0.060	–26.7	–2.3	1.9	–27.2
Li^+^	H_2_O	0.062	0.012	–26.7	0.1	–7.3	–33.9
Na^+^	H_2_O	0.051	0.022	–17.5	0.2	–8.1	–25.4
K^+^	H_2_O	0.039	0.042	–11.0	0.6	–8.5	–19.0
NH_4_^+^	C_4_H_4_S	0.139	0.041	–32.9	–2.5	22.0	–13.4
NH_4_^+^	C_6_H_6_	0.122	0.044	–29.3	–1.9	17.4	–13.8
NH_4_^+^	C_4_H_4_O	0.108	0.041	–27.1	–2.3	17.2	–12.1
NH_4_^+^	C_4_H_4_NH	0.124	0.046	–32.6	–2.4	15.8	–19.2
Li^+^	C_6_H_6_	0.069	0.014	–28.7	–0.3	12.7	–16.4
Na^+^	C_6_H_6_	0.042	0.021	–13.2	–0.1	0.0	–13.3
K^+^	C_6_H_6_	0.047	0.049	–12.8	–0.2	1.4	–11.5
C_6_H_6_	C_6_H_6_	0.080	0.080	–1.8	–1.8	1.2	–2.4
C_5_H_5_N	C_6_H_6_	0.082	0.078	–2.5	–1.8	1.3	–3.0
C_4_H_4_N_2_	C_6_H_6_	0.083	0.078	–3.4	–1.7	2.0	–3.1
DMA	C_6_H_6_	0.089	0.073	–3.8	–1.7	1.5	–4.0
C_6_H_6_	C_6_H_6_ (T)	0.054	0.044	–1.9	–0.9	0.9	–1.9
C_6_H_6_	C_6_H_5_F	0.021	0.015	0.0	0.1	0.5	0.7
C_6_H_6_	C_6_H_5_Cl	0.028	0.060	–0.9	0.1	0.3	–0.5
C_6_H_6_	C_6_H_5_Br	0.099	0.074	–1.2	–0.1	0.2	–1.1
C_6_F_6_	F^–^	0.145	0.046	–6.1	13.2	–21.8	–14.7
C_6_F_6_	Cl^–^	0.187	0.038	–6.5	5.6	–10.3	–11.2
C_6_F_6_	Br^–^	0.099	0.074	–8.0	3.7	–7.2	–11.5

a All the energies are given in kcal/mol. DMA = dimethylacetamide.

**Figure 2:**
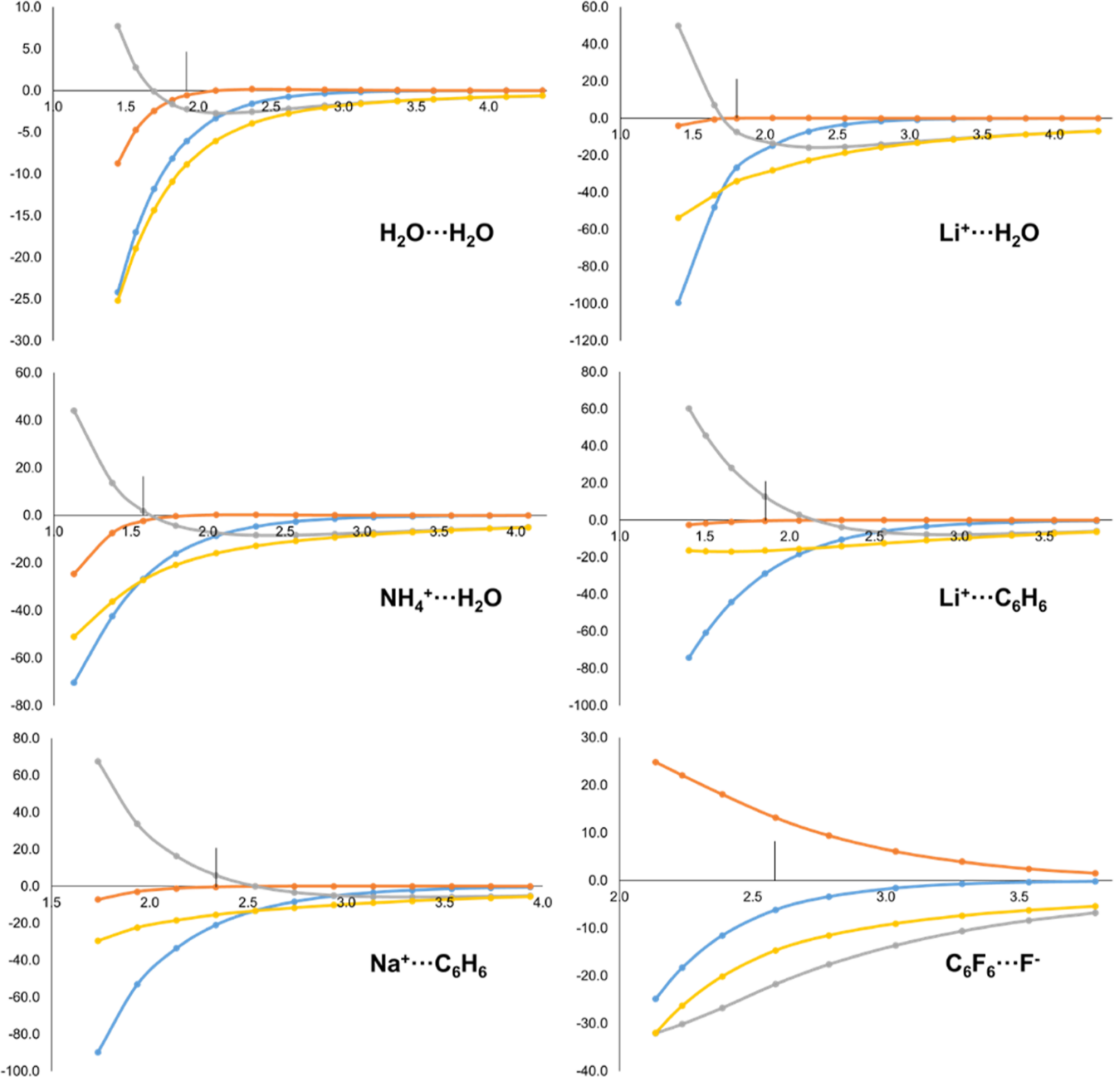
Energy evolution (in kcal/mol, *y*-axis) of Δ*E*_elec_ (yellow) and its IQA-decomposed terms, i.e. Δε_elec,*A*_ (blue), Δε_elec,*B*_ (orange) and Δε_elec,*AB*_ (grey), along the dissociation pathway (in Å, *x*-axis) of H_2_O···H_2_O, Li^+^···H_2_O, NH_4_^+^···H_2_O, Li^+^···C_6_H_6_, Na^+^···C_6_H_6_ and C_6_F_6_···F^–^ molecular systems. Equilibrium distance marked with a vertical line.

To shed light into the origin of these numerical values, we have also considered the evolution of Δ*E*_elec_ as well as its IQA-decomposed terms along the dissociation pathway of representative intermolecular complexes. The results are depicted in Figure 2. As it is well-known, when the frozen densities of the two fragments are brought at the complex’s optimized geometry, Δ*E*_elec_ is favorable^9^ and the shorter the inter-fragment distance, the more negative the total Δ*E*_elec_ contribution. The real-space decomposition of Δ*E*_elec_ yields further insight on this interaction. As previously discussed, the intra-fragment contributions originate from the net electrostatic potential of one fragment interacting with the density of the other fragment that is able to penetrate into its domain. These terms are strongly attractive, particularly in the case of the acceptor *A* (blue curves in Figure 2)*,* as the more ρ_*B*^0^_(**r**) is able to penetrate into the Ω_*A*_ domain, the more negative the Δε_elec,*A*_ contribution becomes. Thus, Δε_elec,*A*_ is enhanced as the interacting fragments come closer in all cases. Furthermore, this contribution is much larger for cationic acceptor species than for neutral ones (notice the different scales in the examples of Figure 2).

In the case of Δε_elec,*B*_ (donor of charge), the trend is similar but the magnitude is much smaller, as the amount of density from the acceptor *A* able to penetrate into the donor is much reduced. In the case of a donor interacting with a hard cation like Li^+^, this term is essentially zero at all interatomic distances (see Li^+^···H_2_O and Li^+^···C_6_H_6_ curves in Figure 2). However, when the donor is anionic, the trend for Δε_elec,*B*_ is completely reversed. Since *N*_*B*^0^_ > Z_*B*_ its *net* electrostatic potential on Ω_*B*_ can be negative, and thus any ρ_*A*^0^_(**r**) able to penetrate into Ω_*B*_ leads to *positive* Δε_elec,*B*_ values. This effect is clearly seen in the C_6_F_6_···F^–^ case of Figure 2.

The usefulness of the IQA decomposition of Δ*E*_elec_ is most clearly seen in the case of Li^+^···C_6_H_6_. As shown in Figure 2, the yellow curve is surprisingly flat, and even becomes *less attractive* at very short distances, totally at odds with the expected behavior. Yet, the overall picture of the intra- and inter-fragment contributions for this system is strikingly similar to that of Li^+^···H_2_O or Na^+^···C_6_H_6_. Close inspection to Figure 2 indicates that the behavior of Δ*E*_elec_ in Li^+^···C_6_H_6_ can be explained by an insufficient enhancement of the intra-fragment contribution of Li^+^ at short distances.

It is worth to note that both Δε_elec,*A*_ and Δε_elec,*B*_ tend asymptotically to zero as the inter-fragment distance increases. This is the expected behavior since at large distances the fragments are essentially in their reference state. Consequently, the inter-fragment Δε_elec,*AB*_ contribution tends to the overall Δ*E*_elec_ value. As the distance decreases, however, Δε_elec,*AB*_ becomes less favorable and even repulsive at very short distances. Thus, the Δε_elec,*AB*_ value for a given complex *at* equilibrium geometry may be slightly positive (e.g. Li^+^···C_6_H_6_) or negative (Li^+^···H_2_O), but the behavior of the components is analogous in both cases.

Still, the Δε_elec,*AB*_ contribution at equilibrium distance is very sensitive to the nature of fragments *A* and *B*. When both *A* and *B* are neutral, the electron-nuclear attraction compensate the nuclear–nuclear repulsion and the Δε_elec,*AB*_ values are very small (ca. ± 2 kcal/mol). However, when the donor *B* is anionic the picture is reversed and at equilibrium Δε_elec,*AB*_ is negative. The case of C_6_F_6_···F^–^ behaves opposite to the other systems (i.e. it becomes more negative as the inter-fragment distance decreases). The second term on the r.h.s. of eq 31 is key to explain this behavior. Since *B* is an anion, ρ_*B*^0^_(**r**) holds an excess of electrons with respect to *Z*_*B*_. In addition, the net potential of *A* in Ω_*B*_ is governed by the nuclear contribution (hence positive). In that scenario, the closer the fragments, the larger the potential and consequently, even if part of ρ_*B*^0^_(**r**) smears into Ω_*A*_, the more dominant the negative term becomes.

**Figure 3:**
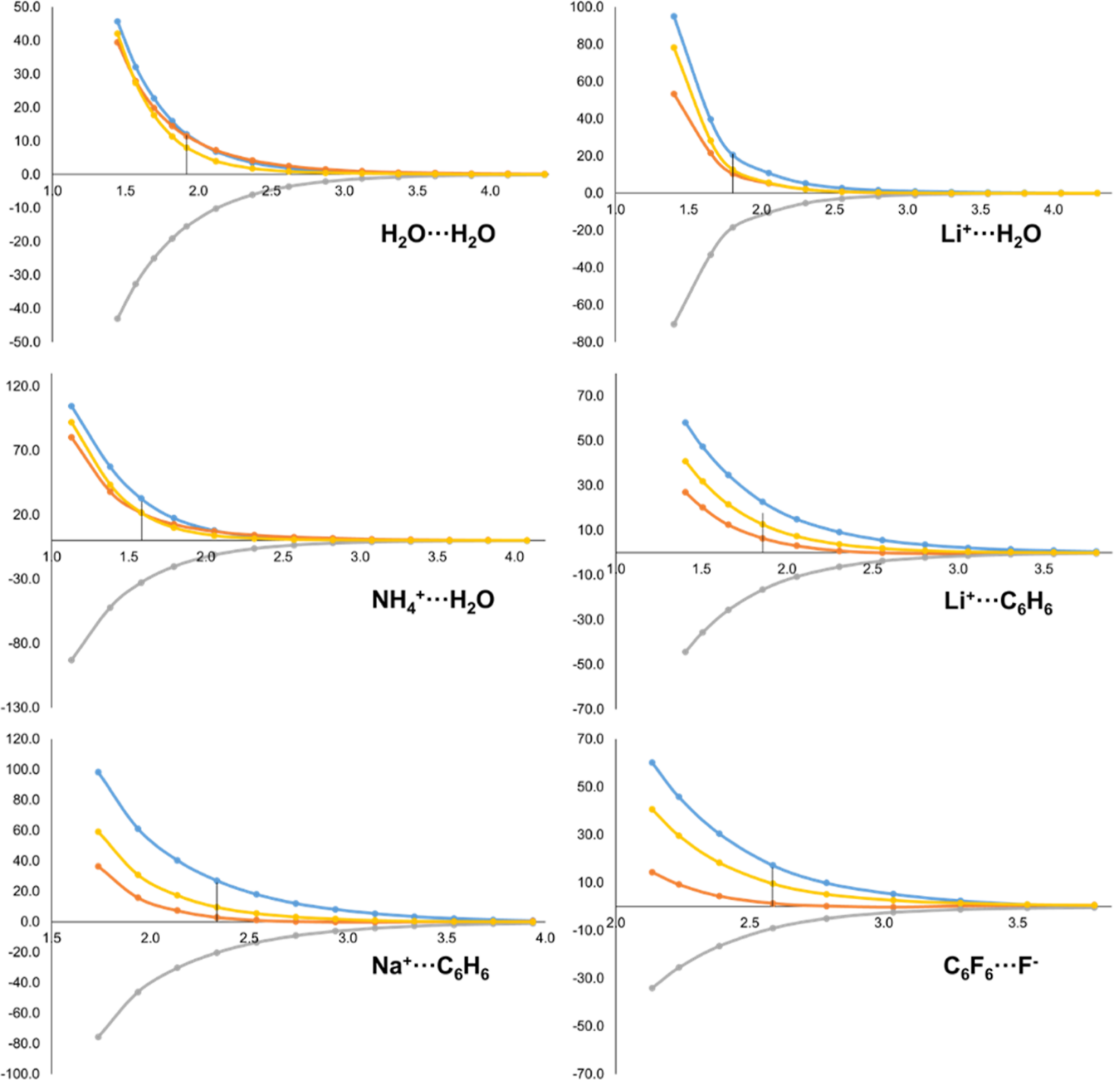
Energy evolution (in kcal/mol, *y*-axis) of Δ*E*_Pauli_ (yellow) and its IQA-decomposed terms, i.e. Δε_Pauli,*A*_ (blue), Δε_Pauli,*B*_ (orange) and Δε_Pauli,*AB*_ (grey), along the dissociation pathway (in Å, *x*-axis) of H_2_O···H_2_O, Li^+^···H_2_O, NH_4_^+^···H_2_O, Li^+^···C_6_H_6_, Na^+^···C_6_H_6_ and C_6_F_6_···F^–^ molecular systems. Equilibrium distance marked with a vertical line.

Let us proceed by analyzing the Pauli repulsion EDA term, Δ*E*_Pauli_, whose contribution originates from the intermediate state *A*^0^*B*^0^. Bickelhaupt and Baerends showed that the antisymmetrization of the frozen fragment densities to build *A*^0^*B*^0^ induces an electron density flow from the intermolecular region to the atomic regions.^9^ By decomposing Δ*E*_Pauli_ into kinetic (Δ*T*^0^) and potential (Δ*V*_Pauli_) terms, they showed that the contraction effect translates into an increase of the kinetic energy, and a concomitant decrease (more negative) of the potential energy. The latter is due to the fact that more density is accumulated at regions (e.g. close to nuclei) where the Coulombic potential is larger. The IQA decomposition of Δ*E*_Pauli_ recovers this picture from a real-space perspective. By definition, kinetic energy contributions only have intra-fragment character upon IQA decomposition and, consequently, they are captured by the Δε_Pauli,*A*_ and Δε_Pauli,*B*_ terms. In other words, the Δε_Pauli,*AB*_ term solely contains potential energy contributions. The kinetic energy increase is so dominant that these terms are expected to be positive and increase along the shortening of the inter-fragment distance. This is exactly the behavior depicted in Figure 3. Furthermore, the corresponding values at equilibrium distance (Table 2) indicate positive contributions for both the donor and the acceptor fragments with very few exceptions.

**Table 2:** Fragment (IQA) Decomposition of the Δ*E*_Pauli_ Term from EDA of the Systems Studied^a^

A = acceptor	B = donor	Δε_Pauli,*A*_	Δε_Pauli,*B*_	Δε_Pauli,*AB*_	Δ*E*_Pauli_
H_2_O	H_2_O	12.0	11.5	–15.5	8.0
H_2_O	MeOH	14.2	12.5	–17.2	9.5
MeOH	MeOH	14.3	14.0	–18.0	10.2
H_2_O	NH_3_	15.6	15.1	–18.5	12.2
NH_4_^+^	H_2_O	32.8	21.7	–32.7	21.8
Li^+^	H_2_O	20.6	10.5	–18.4	12.6
Na^+^	H_2_O	22.4	5.6	–19.7	8.3
K^+^	H_2_O	27.7	1.3	–22.2	6.7
NH_4_^+^	C_4_H_4_S	16.2	26.4	–27.2	15.4
NH_4_^+^	C_6_H_6_	17.5	23.6	–26.5	14.6
NH_4_^+^	C_4_H_4_O	17.0	22.3	–24.8	14.4
NH_4_^+^	C_4_H_4_NH	21.9	24.6	–27.7	18.8
Li^+^	C_6_H_6_	22.7	6.5	–16.5	12.7
Na^+^	C_6_H_6_	18.0	1.1	–13.5	5.6
K^+^	C_6_H_6_	30.5	–1.8	–21.7	7.0
C_6_H_6_	C_6_H_6_	10.5	10.5	–13.9	7.1
C_5_H_5_N	C_6_H_6_	11.8	10.0	–14.4	7.4
C_4_H_4_N_2_	C_6_H_6_	12.4	9.6	–14.9	7.1
DMA	C_6_H_6_	11.5	12.0	–16.3	7.2
C_6_H_6_	C_6_H_6_ (T)	5.8	7.8	–9.6	4.1
C_6_H_6_	C_6_H_5_F	2.1	2.5	–3.5	1.2
C_6_H_6_	C_6_H_5_Cl	13.8	–2.0	–9.2	2.7
C_6_H_6_	C_6_H_5_Br	18.0	–2.9	–11.8	3.3
C_6_F_6_	F^–^	17.2	1.4	–9.0	9.6
C_6_F_6_	Cl^–^	6.3	7.0	–5.1	8.3
C_6_F_6_	Br^–^	3.8	11.0	–5.5	9.2

a All the energies are given in kcal/mol. DMA = dimethylacetamide.

On the other hand, the Δε_Pauli,*AB*_ contributions are large and negative in all cases, and also become more favorable at shorter distances. The origin of this behavior is that, according to eq 33, this term does not explicitly contain energy differences between the intermediate and isolated fragment’s states, as there is no inter-fragment term associate to the latter. Deeper analysis indicates that the classical part of the potential energy differences cancels (particularly in the neutral complexes), so the inter-fragment exchange–correlation contribution becomes the dominant term.

Note that the aforementioned contraction effect also increases (becomes more negative) the overall exchange–correlation energy of *A*^0^*B*^0^ with respect to that of *A*^0^ and *B*^0^. The dominant exchange contribution is governed by the density close to the nuclei, by virtue of its *ρ*(***r***)^4/3^ dependence. It might appear counterintuitive that a charge depletion in the inter-atomic region leads, nevertheless, to a negative inter-fragment exchange–correlation. As pointed out by Salvador and Mayer, neither the bond order nor the Hartree–Fock exchange energy components are directly related to overlap populations, but to part of the density localized on the atoms that leads to a correlation between the fluctuations of the atomic populations, even in the absence of overlap.^25^ Indeed, inter-atomic exchange energy contribution in the Salvador–Mayer KS-DFT IQA formulation originates on the bond order density between a pair of atoms, which is actually *large* in the vicinity of the nuclei.^25^ An even simpler explanation is that part of the exchange–correlation energy of the *A*^0^*B*^0^ state is assigned to inter-fragment character by the IQA decomposition, while, once again, there is no inter-fragment contribution from the isolated fragments to compensate for it, as shown in eq 33.

The net result (see Table 2) is that the Δε_Pauli,*AB*_ contributions are systematically large and negative. On the contrary, the intra-fragment Δε_Pauli,*A*_ and Δε_Pauli,*B*_ terms are positive, mimicking the behavior of Δ*T*^0^, but bearing not just kinetic but also intra-fragment electrostatic and exchange–correlation contributions. Beyond the overall trend, it is not easy to compare the values of the inter- and intra-fragment contributions from one system to another, especially among different interaction types. Again, even though the behavior of the IQA components is analogous for all interaction types, the actual numerical values are largely dictated by the respective equilibrium distances.

**Figure 4:**
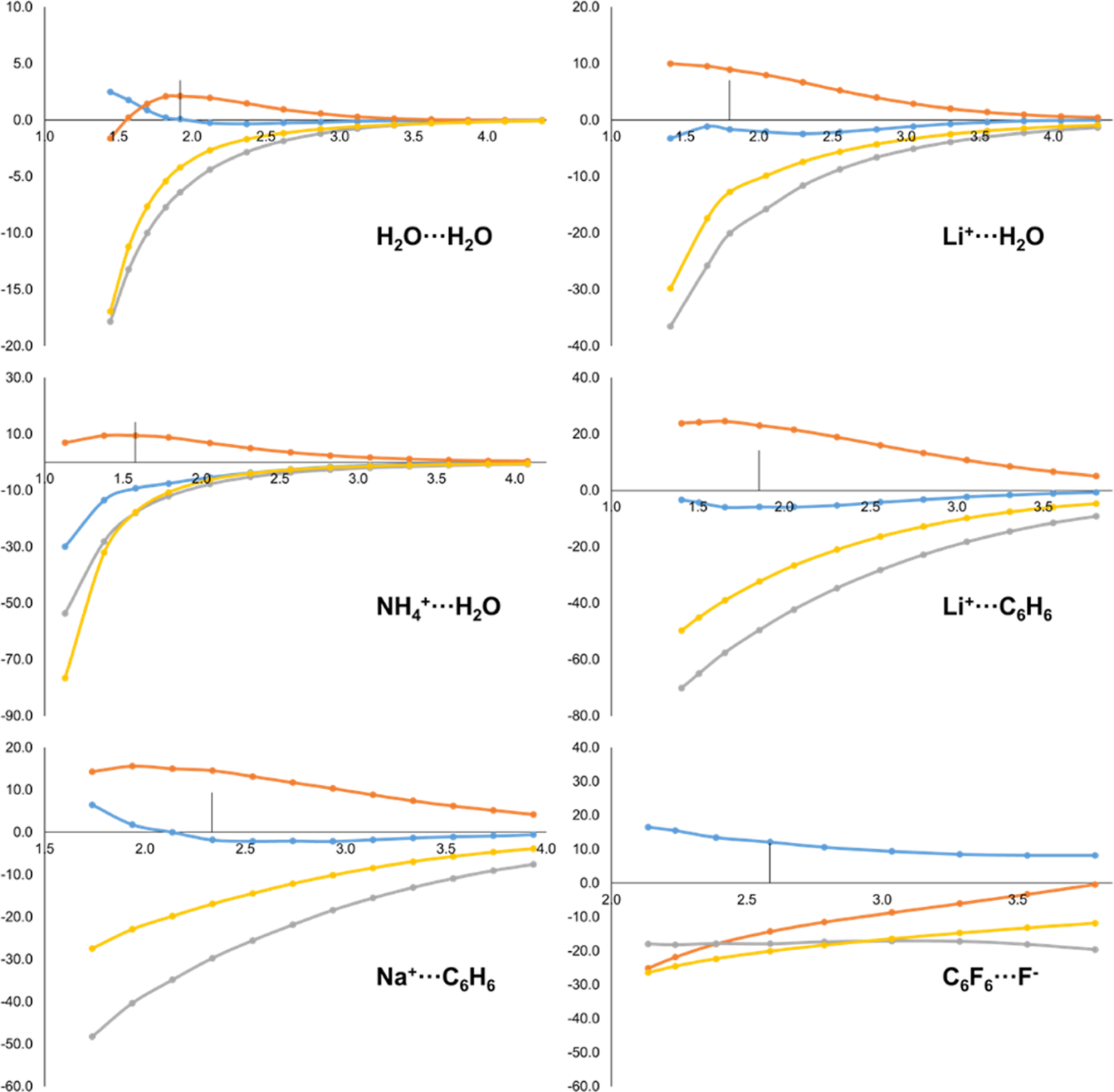
Energy evolution (in kcal/mol, *y*-axis) of Δ*E*_orb_ (yellow) and its IQA-decomposed terms, i.e. Δε_orb,*A*_ (blue), Δε_orb,*B*_ (orange) and Δε_orb,*AB*_ (grey), along the dissociation pathway (in Å, *x*-axis) of H_2_O···H_2_O, Li^+^···H_2_O, NH_4_^+^···H_2_O, Li^+^···C_6_H_6_, Na^+^···C_6_H_6_ and C_6_F_6_···F^–^ molecular systems. Equilibrium distance marked with a vertical line.

The orbital interaction from EDA, Δ*E*_orb_, originates from the relaxation of the MOs of the complex’s intermediate state *A*^0^*B*^0^ to the final complex’s *AB* ground state. It is by definition a negative contribution (if the final state of *AB* is the ground state), that compensates for the repulsive Pauli term. At short intermolecular distances the intermediate state *A*^0^*B*^0^ is higher in energy, so that the relaxation energy to the state *AB* becomes more negative. This behavior can be observed in Figure 4 (yellow curve) for all systems. The orbital relaxation induces an increase of electron density in the inter-atomic (and thus intermolecular) region, making the inter-fragment exchange–correlation contributions of the *AB* ground state *larger* (in absolute value) compared to the ones from the intermediate state *A*^0^*B*^0^. This is captured by the Δε_orb,*AB*_ term (grey curve in Figure 4), that closely follows the trend of the global Δ*E*_orb_ value, with the exception of the C_6_F_6_···F^–^ system but for reasons that will be disclosed later. The trends observed for the intra-fragment terms (blue and orange curves) vary according to the nature of the donor and acceptor moieties. The intra-fragment contribution of the electron donor, Δε_orb,*B*_, vanishes at long distances but as the fragments approach it becomes destabilizing. At distances much shorter than the equilibrium the term becomes less repulsive and can even be stabilizing in the case of the water dimer. On the contrary, the intra-fragment contribution for the acceptor, Δε_orb,*A*_, is very small (particularly at equilibrium distances) but usually stabilizing along the dissociation profile.

The decomposition of Δ*E*_orb_ at the equilibrium geometries can be found in Table 3. It is well-known that the Δ*E*_orb_ contribution accounts for both polarization and charge-transfer effects from the intermediate to the final state. It is precisely the amount of charge-transfer that largely dominates these intra-fragment contributions to Δ*E*_orb_. The more charge is transferred to the acceptor *A* going from the intermediate *A*^0^*B*^0^ state to the final state, the more stabilizing the Δε_orb,*A*_ contribution, as shown in Figure S1 of the Supporting Information. In the case of the donor moieties the correlation is not as good, but the contributions follow the same trend: the more charge is transferred to the acceptor, the more destabilizing the Δε_orb,*B*_ values are.

**Table 3:** Fragment (IQA) Decomposition of the Δ*E*_orb_ Term from EDA of the Systems Studied^a^

A = acceptor	B = donor	Δε_orb,*A*_	Δε_orb,*B*_	Δε_orb,*AB*_	Δ*E*_orb_
H_2_O	H_2_O	0.1	2.1	–6.4	–4.2
H_2_O	MeOH	0.5	2.1	–7.8	–5.3
MeOH	MeOH	0.3	2.1	–8.0	–5.6
H_2_O	NH_3_	–0.2	2.5	–8.5	–6.1
NH_4_^+^	H_2_O	–9.3	9.4	–17.9	–17.7
Li^+^	H_2_O	–1.6	8.9	–20.0	–12.7
Na^+^	H_2_O	–0.7	5.6	–11.4	–6.5
K^+^	H_2_O	–1.5	4.4	–7.9	–5.0
NH_4_^+^	C_4_H_4_S	–16.4	21.4	–23.9	–18.9
NH_4_^+^	C_6_H_6_	–14.7	17.2	–20.1	–17.6
NH_4_^+^	C_4_H_4_O	–15.2	16.5	–19.1	–17.7
NH_4_^+^	C_4_H_4_NH	–18.1	18.0	–21.8	–22.0
Li^+^	C_6_H_6_	–5.8	23.1	–49.6	–32.3
Na^+^	C_6_H_6_	–2.1	13.2	–25.5	–14.4
K^+^	C_6_H_6_	–2.0	9.9	–18.8	–10.9
C_6_H_6_	C_6_H_6_	0.4	0.4	–2.1	–1.3
C_5_H_5_N	C_6_H_6_	0.2	0.8	–2.5	–1.5
C_4_H_4_N_2_	C_6_H_6_	–0.1	1.4	–3.0	–1.7
DMA	C_6_H_6_	0.2	1.2	–3.4	–2.0
C_6_H_6_	C_6_H_6_ (T)	–0.1	0.8	–1.8	–1.2
C_6_H_6_	C_6_H_5_F	0.0	0.0	–0.5	–0.4
C_6_H_6_	C_6_H_5_Cl	0.0	0.5	–1.2	–0.7
C_6_H_6_	C_6_H_5_Br	0.1	0.6	–1.5	–0.8
C_6_F_6_	F^–^	12.0	–14.2	–17.9	–20.1
C_6_F_6_	Cl^–^	4.4	–4.2	–10.7	–10.5
C_6_F_6_	Br^–^	2.3	–1.2	–9.1	–8.0

a All the energies are given in kcal/mol. DMA = dimethylacetamide.

Remarkably, the anion−π systems exhibit an opposite trend. The anion donates charge upon interaction, yet the Δε_orb,*B*_ contribution is stabilizing. This holds along the whole dissociation profile, as shown in Figure 4. At the same time, the acceptor gains charge but its Δε_orb,*A*_ contribution is destabilizing. One can also note in Figure 4, the wrong asymptotics of the intra- and inter-fragment contributions for C_6_F_6_···F^–^ at long distances. This is in fact a clear fingerprint of delocalization error in the KS density, coming from the BP86 functional. First, the dissociation profile could not be further extended at longer distances due to severe SCF convergence problems but, most importantly, the partial charge on F^–^ actually *increases* from a value of −0.841 at 3.79 Å distance to −0.865 at equilibrium distance, which might explain the aforementioned opposite trend of these systems. It is beyond the scope of the present work to examine the dependence of the decomposed terms on the underlying density functional approximation, but it appears the chosen level of theory is not particularly appropriate to describe these anion-π interactions.

Of course, since the present EDA–IQA decomposition is fully additive, one can obtain the intra- and inter-fragment decomposition of the total interaction energy, Δ*E*_int_, by adding the corresponding electrostatic, Pauli repulsion and orbital interaction terms (and dispersion, if included). Numerically, this is not necessary as one can simply perform a conventional IQA decomposition of the final *AB* state of the complex and subtract the isolated fragment’s energies of *A*^*0*^ and *B*^*0*^ to obtain the intra-fragment or deformation contributions.

**Figure 5:**
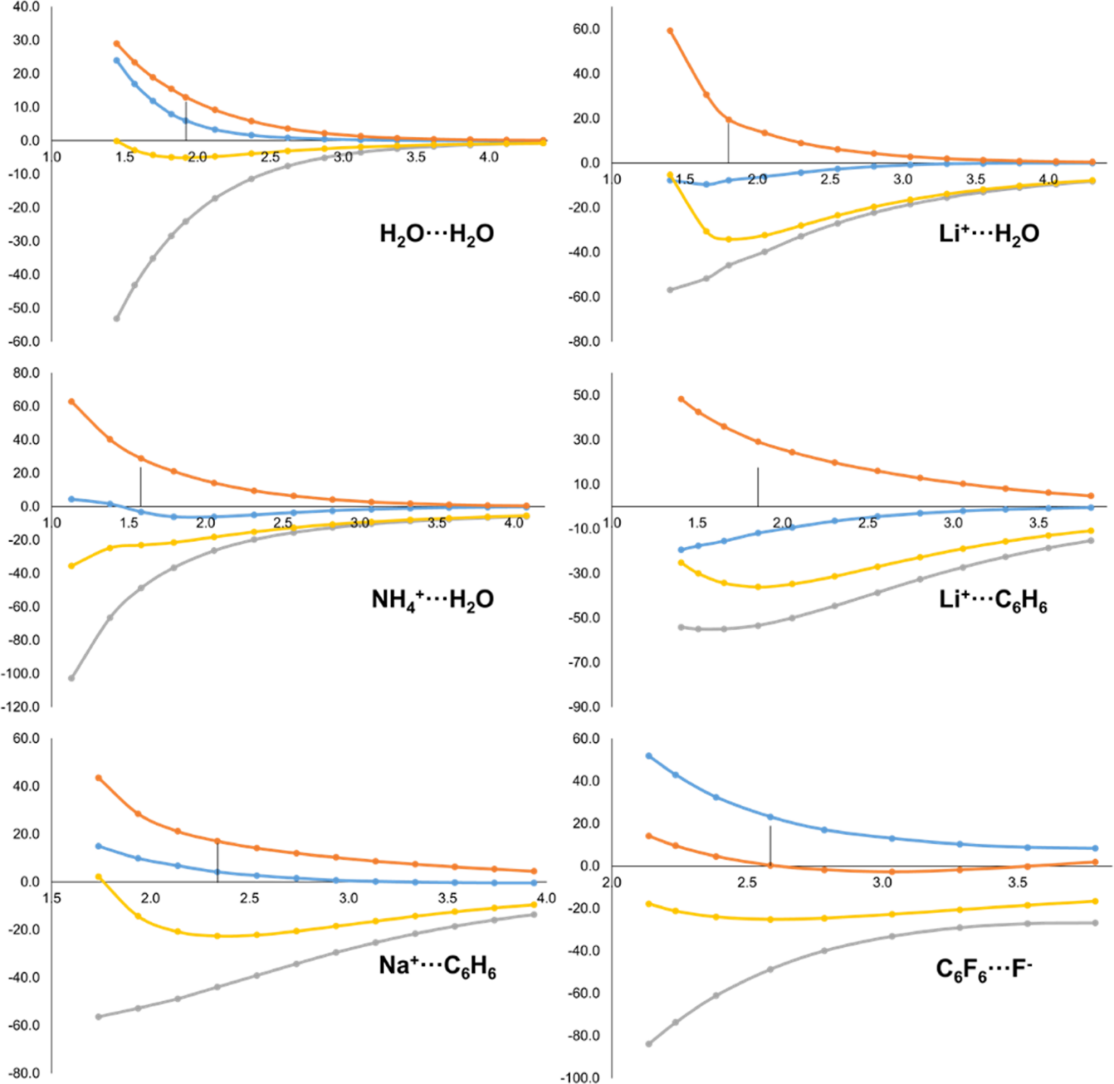
Energy evolution (in kcal/mol, *y*-axis) of Δ*E*_int_ (yellow) and its IQA-decomposed terms, i.e. Δε_def.el,*A*_ (blue) and Δε_def.el,*A*_ (orange) and Δε_int,*AB*_ (grey), along the dissociation pathway (in Å, *x*-axis) of H_2_O···H_2_O, Li^+^···H_2_O, NH_4_^+^···H_2_O, Li^+^···C_6_H_6_, Na^+^···C_6_H_6_ and C_6_F_6_···F^–^ molecular systems. Equilibrium distance marked with a vertical line.

For completeness, the IQA decomposition of Δ*E*_int_ along the dissociation profile of the representative systems is shown in Figure 5, while the corresponding values at equilibrium geometries are gathered on Table 4. Similarly to the orbital interaction contribution, the electronic deformation energies (Δε_def.el,*A*_ and Δε_def.el,*B*_) at equilibrium are governed by the amount of charge transfer, in this case between the final state and the that of the isolated free fragments. Note that this charge-transfer is different from the one accounted for in the orbital interaction term because, in real-space analysis, there is already some charge-transfer when forming the intermediate *A*^0^*B*^0^ state. Since the charge transfer from the isolated fragments to the final state is larger, the electronic deformation energies on Table 4 are larger (in absolute value) as well. The correlation between the electronic deformation energies of both the donor and acceptor moieties and the respective amount of charge-transfer is excellent (*r*^2^ = 0.95, see Figure S2 of the Supporting Information). However, the correlation curve does not cross the (0,0) point but slightly above. That is, even though the acceptor *A* can eventually gain a small amount of charge (e.g. 0.05e for Na^+^ in Na^+^···C_6_H_6_), the corresponding electronic deformation energy is still slightly positive (+2.7 kcal/mol), due to the accompanying polarization of the fragment’s density within the complex. Finally, as usual in the conventional IQA analysis, the Δε_int,*AB*_ contributions are largely stabilizing along the dissociation profile and also at equilibrium, even for the dispersion–bound complexes (notice that the interaction energies in Table 4 do not contain the dispersion correction). There is also a decent correlation (*r*^2^ = 0.82, see Figure S3 of the Supporting Information) between the Δε_int,*AB*_ and Δ*E*_int_ values at equilibrium, even considering the unreliable anion-π complexes.

**Table 4:** Fragment (IQA) Decomposition of the Δ*E*_int_ Term from EDA of the Systems Studied^a^

A = acceptor	B = donor	Δε_def.el,*A*_	Δε_def.el,*A*_	Δε_int,*AB*_	Δ*E*_int_
H_2_O	H_2_O	6.0	13.1	–24.1	–5.0
H_2_O	MeOH	7.8	13.7	–26.7	–5.2
MeOH	MeOH	7.3	15.0	–27.4	–5.1
H_2_O	NH_3_	5.2	17.2	–29.3	–6.8
NH_4_^+^	H_2_O	–3.2	28.8	–48.8	–23.1
Li^+^	H_2_O	–7.7	19.5	–45.8	–34.0
Na^+^	H_2_O	4.2	11.5	–39.2	–23.6
K^+^	H_2_O	15.1	6.3	–38.6	–17.2
NH_4_^+^	C_4_H_4_S	–33.1	45.4	–29.2	–16.9
NH_4_^+^	C_6_H_6_	–26.5	38.8	–29.2	–16.8
NH_4_^+^	C_4_H_4_O	–25.2	36.5	–26.7	–15.4
NH_4_^+^	C_4_H_4_NH	–28.8	40.2	–33.7	–22.4
Li^+^	C_6_H_6_	–11.9	29.2	–53.4	–36.1
Na^+^	C_6_H_6_	2.7	14.2	–39.1	–22.2
K^+^	C_6_H_6_	15.8	7.8	–39.1	–15.5
C_6_H_6_	C_6_H_6_	9.1	9.1	–14.8	7.0
C_5_H_5_N	C_6_H_6_	9.4	9.0	–15.6	7.2
C_4_H_4_N_2_	C_6_H_6_	8.9	9.4	–16.0	7.3
DMA	C_6_H_6_	7.9	11.5	–18.2	6.7
C_6_H_6_	C_6_H_6_ (T)	3.8	7.7	–10.5	3.7
C_6_H_6_	C_6_H_5_F	2.1	2.7	–3.4	1.4
C_6_H_6_	C_6_H_5_Cl	12.9	–1.4	–10.1	1.4
C_6_H_6_	C_6_H_5_Br	16.8	–2.4	–13.1	1.3
C_6_F_6_	F^–^	23.1	0.3	–48.7	–25.2
C_6_F_6_	Cl^–^	4.2	8.4	–26.1	–13.5
C_6_F_6_	Br^–^	–1.9	13.5	–21.9	–10.2

a All the energies are given in kcal/mol. DMA = dimethylacetamide.

So far, we have presented cases where the EDA-IQA terms are conveniently grouped to match the fragments selected in the EDA step straightly. However, the pairwise nature of the IQA terms can also be used to identify the directionality of each specific interaction. As an example, we have chosen the series of lithium carbanions: LiCF_3_, LiCHF_2_, LiCH_2_F, and LiCH_3_. For the sake of comparison, we have enforced the symmetry of all species, so that not all structures correspond to a minimum in the PES. Table 5 summarizes the EDA results for the Li–C bond using the ionic reference fragments Li^+^ (^1^S) and CR_3_^–^ (^1^A_1_).

**Table 5:** EDA–IQA Results of LiCF_3_, LiCHF_2_, LiCH_2_F, and LiCH_3_ at the BP86-D3(BJ)/def2-TZVPP Level of Theory

	LiCF_3_	*LiCHF*_*2*_*(forced)*	*LiCH*_*2*_*F (forced)*	LiCH_3_
Δ*E*_int_	–154.3	*–158.7*	*–171.3*	–179.2
Δε_int,Li_	–23.2	–20.8	–26.1	–28.7
Δε_int,CR3_	10.1	9.2	5.6	4.8
Δε_int,Li–CR3_	–141	–147.2	–150.8	–155.3
Δε_int,Li–C_	124.8	10.9	–39.9	–107.5
Δε_int,Li–F_	–88.6	–76.0	–85.0	
Δε_int,Li–H_		–6.1	–12.9	–15.9
Δ*E*_Pauli_	35.2	*36.6*	*46.4*	51.4
Δε_Pauli,Li_	45.6	48.3	62.2	68.2
Δε_Pauli,CR3_	7.2	5.5	2.2	0.2
Δε_Pauli,Li–CR3_	–17.6	–17.2	–18.0	–17.0
Δε_Pauli,Li–C_	–66.4	–45.5	–43.8	–30.4
Δε_Pauli,Li–F_	16.3	13.3	18.4	
Δε_Pauli,Li–H_		1.8	3.7	4.5
Δ*E*_elec_	–170.1	*–174.6*	*–195.0*	–206.5
Δε_elec,Li_	–68.2	–67.7	–83.7	–90.9
Δε_elec,CR3_	1.4	1.4	1.5	1.6
Δε_elec,Li–CR3_	–103.2	–108.4	–112.8	–117.1
Δε_elec,Li–C_	244.0	108.9	58.0	–21.7
Δε_elec,Li–F_	–115.8	–98.8	–114.0	
Δε_elec,Li–H_		–19.8	–28.4	–31.8
Δ*E*_orb_	–19.3	*–20.7*	*–22.7*	–24.1
Δε_orb,Li_	–0.6	–1.4	–4.6	–6.0
Δε_orb,CR3_	1.5	2.3	1.9	3.0
Δε_orb,Li–CR3_	–20.2	–21.6	–20	–21.2
Δε_orb,Li–C_	–52.8	–52.5	–54.1	–55.4
Δε_orb,Li–F_	10.9	9.5	10.6	
Δε_orb,Li–H_		11.9	11.8	11.4
Δ*E*_disp_	–1.0	*–1.0*	*–1.0*	–1.0
Δ*E*_prep_	3.9	*1.6*	*1.4*	0.3
*d*(Li–C)	1.999	*1.999*	*1.999*	1.977

The dissociation energy values show that the more hydrogen atoms are in the molecule, the stronger the Li-CR_3_ bond is. The interaction energy follows the same trend, as the preparation energy represents small energy penalties upon deformation. The nature of the chemical bond from the reference fragments is mainly ionic, since the electrostatic interaction represents between 85 and 90% of the total stabilizing interactions. All Pauli repulsion, electrostatic and orbital interaction terms increase in absolute value going from LiCF_3_ to LiCH_3_. Applying EDA-IQA to the series brings further insight into the reason behind these trends. The results are gathered in Table 5. The IQA decomposition of ΔE_elec_ shows a large stabilization of the Li^+^ moiety, that increases along the series. As discussed before, this stabilization originates from the CR_3_^–^ density penetration into the Li^+^ domain. The presence of highly electronegative F atoms in the CF_3_^–^ moiety reduces the electron density at the carbon atom, which is closer in space, compared to the less electronegative H atoms in CH_3_^–^. With the frozen densities of the CR_3_^–^ and Li^+^ fragments interact, the more H atoms the more charge penetration into the Li^+^ domain, resulting in an enhanced Δε_elec,Li_ contribution. The inter-fragment Δε_elec,Li–CR3_ contribution is also stabilizing, an also increases along the series. Further IQA decomposition into atomic and diatomic contributions helps to rationalize the trend. The contribution of each Li···F interaction to the electrostatic term (ca. 110–115 kcal/mol) is significantly larger than the contribution of the less ionic Li···H counterparts (ca. 20–30 kcal/mol). However, it is the direct Li–C interaction that is most affected by the nature of the substituent R. While this term is largely destabilizing in LiCF_3_, cancelling out to some extent the Li···F interactions, it is even slightly stabilizing in LiCH_3_. The EDA-IQA decomposition provides actual quantification for the qualitative argument that an electron deficient C atom (like in CF_3_^–^) will exhibit electrostatic repulsion with the cationic Li^+^ moiety.

The orbital relaxation represents only ca. 10% of the total attractive interactions. Its IQA decomposition reveals that the leading term is the inter-fragment Δε_orb,Li-CR3_, but the trend is explained by the contribution of the Li^+^ fragment. The orbital interaction term gathers both charge-transfer and polarization effects. When using charged reference fragments, like in this case, charge transfer should dominate, because the ionicity of the final state is reduced upon bond formation. Going from LiCF_3_ to LiCH_3_ species the bond ionicity is reduced, hence more charge is transferred to the Li^+^ fragment, enhancing its contribution from −0.6 to −6 kcal/mol, respectively. The inter-fragment contribution to the Δ*E*_orb_ is still the leading term, but it remains essentially constant along the series, because the orbital relaxation effects are essentially the same for the Li···F and Li···H contacts.

Regarding to the Pauli repulsion EDA term, its total value monotonically increases from Li^+^···CF_3_^–^ (35.2 kcal/mol) to Li^+^···CH_3_^–^ (51.4 kcal/mol). As previously discussed, the highly repulsive kinetic energy contribution is gathered by the atomic EDA-IQA terms, while the stabilizing potential energy contribution spreads more importantly over the diatomic terms. In this case, the trend along the series is better captured by the highly repulsive contribution of Li^+^. The less electron rich C atom of CF_3_^–^ leads to a decreased electron reorganization on the Li^+^ fragment upon orthogonalization and antisymetrization to build the intermediate state. On the other hand, it is interesting to note the difference between the contributions of the interatomic Li···C and Li···H/F terms. The former is strongly stabilizing, because the orthogonalization and antisymmetrization affects to a larger extent the inter-fragment region (i.e. the Li–C σ-bond). On the contrary, the same electron reorganization weakens the Li···H and Li···F diatomic terms, and their contribution to the Pauli term is repulsive. The balance of these interatomic contributions makes the inter-fragment contribution to the Pauli repulsion indeed favorable but almost along the series.

Finally, it is worth to note that the atomic or group contributions of the EDA terms are numerically affected by the particular shape of the atomic weight functions. It is known, albeit not much discussed in the literature, that in the case of Hirshfeld-type approaches the values of the atomic weight functions of light atoms on top of the nucleus is not exactly one, as it is the case for QTAIM or TFVC schemes. For instance, the weight of C atom on top of each H nucleus in CH_4_ using conventional Hirshfeld scheme is ca. 0.1.^26^ Hence, the charge penetration predicted by Hirshfeld-type schemes can be much larger. Charge penetration is not too significant in terms of electron population/charge, as it is very small compared to the overall atomic density. However, its effect on the energetics of the electrostatic term (i.e. eqns. 30 and 31) can be much more relevant, as the nuclear position is precisely where the nuclear potential is larger. Table S3 of the Supporting Information gathers the results obtained for some hydrogen-bonded and ion-dipole systems using atomic weight functions from the Hirshfeld-Iterative^32^ (HI) scheme. Charge-penetration effects on the electrostatic term are mostly captured by the Δε_elec,*AB*_ contribution. While TFVC and HI yield similar values for the water dimer and water–methanol systems, this contribution is clearly enhanced for the ionic systems Li^+^···H_2_O or Na^+^···H_2_O.

However, charge penetration effects are even more dramatic in the case of Pauli repulsion, again particularly for the charged species. In this case, it originates from the fact that the individual terms are obtained by differences between IQA energies of the isolated fragments and those of the molecular complex, where the fragments share the physical space. Such interprenetration is much more significant using HI and as a consequence, Δ_Paulil,Li_ for Li^+^···H_2_O changes from +20.6 kcal/mol using TFVC to up to 72.1 kcal/mol using HI. On the other hand, the decomposition of the orbital interaction contribution leads to very similar results for both TFVC and HI, precisely because the terms are obtained by comparing IQA energies of the molecular complex at the final and intermediate state (i.e. in both cases the fragments share the physical space). Adding up all EDA terms obtained with HI leads to a final EDA-IQA decomposition of the interaction energy with much larger intra- and inter-fragment contributions of different sign that compensate each other. For this reason, we do not recommend the use of Hirshfeld-type schemes for the present EDA-IQA scheme.

## Conclusions

In this work, we have presented the implementation of the IQA decomposition of the individual terms arising from the Kitaura-Morokuma (KM) EDA methodology, namely electrostatic, Pauli repulsion, and orbital interaction. The EDA-IQA approach has been illustrated for a set of complexes, covering different types of intermolecular interactions. In this context, the atomic and diatomic contributions obtained for each EDA term have been conveniently grouped into intra-fragment and inter-fragment terms. Although the EDA-IQA terms can be grouped to match the selected fragments, the methodology affords atomic and pairwise interactions between all atoms of the system in all EDA terms, helping the precise identification of ruling effects. This in-depth analysis affords a better rationalization of the trends of the bonding along the LiCF_3_ to LiCH_3_ series. Through the lens of real-space analysis such as IQA, the electrostatic interaction from EDA can no longer be seen as intermolecular in nature, but also results in meaningful and non-negligible intra-fragment contributions, because the interacting fragments share the physical space once the complex is formed. The EDA-IQA decomposition of the Pauli repulsion shows destabilizing intra-fragment contributions, particularly in the case of fragments that are net charge acceptors. On the contrary, the inter-fragment Pauli contribution is strongly stabilizing. The intra- and inter-fragment Δ*E*_Pauli_ contributions closely mimic the behavior of the classical decomposition of Pauli repulsion into kinetic and potential terms, respectively. In the case of the orbital interaction term, the sign and magnitude of the intra-fragment contribution at equilibrium geometries is largely driven by the amount of charge transfer: the net acceptors of charge stabilize and the donor moieties destabilize. The proper asymptotics profile of all EDA-IQA terms is also confirmed along the intermolecular dissociation path. Finally, while this work focuses on the particular implementation of (KM) EDA-IQA analysis for intermolecular interactions, it can be readily applied to other EDA schemes relying in intermediate states such as ALMO-EDA.

## Supporting Information Available

The Supporting Information contains the correlations between the intra-fragment orbital interaction and interaction energy components (IQA), with the fragment charge differences associated to their associated states (Figures S1 and S2, respectively), the correlation between the total interaction energy (EDA) and its inter-fragment (IQA) component (Figure S3), fragment charges (TFVC) for each of the states of the EDA process (Table S1), fragment charge differences (TFVC) associated to each EDA energy term (Table S2) and a comparison of TFVC and Hirshfeld-Iterative EDA-IQA results for selected systems (Table S3)(PDF)
Coordinates of the studied systems (XYZ format) (XYZ)

## Supplementary Material

ct3c00143_si_001.pdf

ct3c00143_si_002.xyz
