# Supplementary material for: Merging the Energy Decomposition Analysis with the Interacting Quantum Atoms Approach
Source: J Chem Theory Comput. 2023 May 29;19(12):3469–85. doi: 10.1021/acs.jctc.3c00143 (PMC10308825; doi:10.1021/acs.jctc.3c00143)
Supplement: Supplementary file 1 — ct3c00143_si_001.pdf [file ct3c00143_si_001.pdf]

Supporting Information for: “Merging the Energy Decomposition Analysis and the Interacting Quantum Atoms Approaches”

Martí Gimferrer,[a] Sergi Danés,[a,b] Diego M. Andrada,[b] Pedro Salvador.[a,]*

a) Institut de Química Computacional i Catàlisi i Departament de Química, Universitat de Girona, c/ Maria Aurèlia Capmany i Farnés 69, 17003, Girona, Catalonia, Spain.

b) Faculty of Natural Sciences and Technology, Department of Chemistry, Saarland University, 66123 Saarbrücken, Federal Republic of Germany.

Email: pedro.salvador@udg.edu

TABLE OF CONTENTS

Figures S1-S2. Correlations between the fragment IQA energy components and the charge differences associated to the corresponding EDA energy component.

Figure S3. Correlation between the total interaction energy (EDA) and its inter-fragment (IQA) decomposed quantity.

Table S1. Fragment charges (TFVC) for each of the states of the EDA process.

Table S2. Fragment charge differences (TFVC) associated to each energy term of the EDA process.

Table S3. Comparison of the fragment (IQA) decomposition of each energy term from EDA for some selected systems using different atomic definitions.

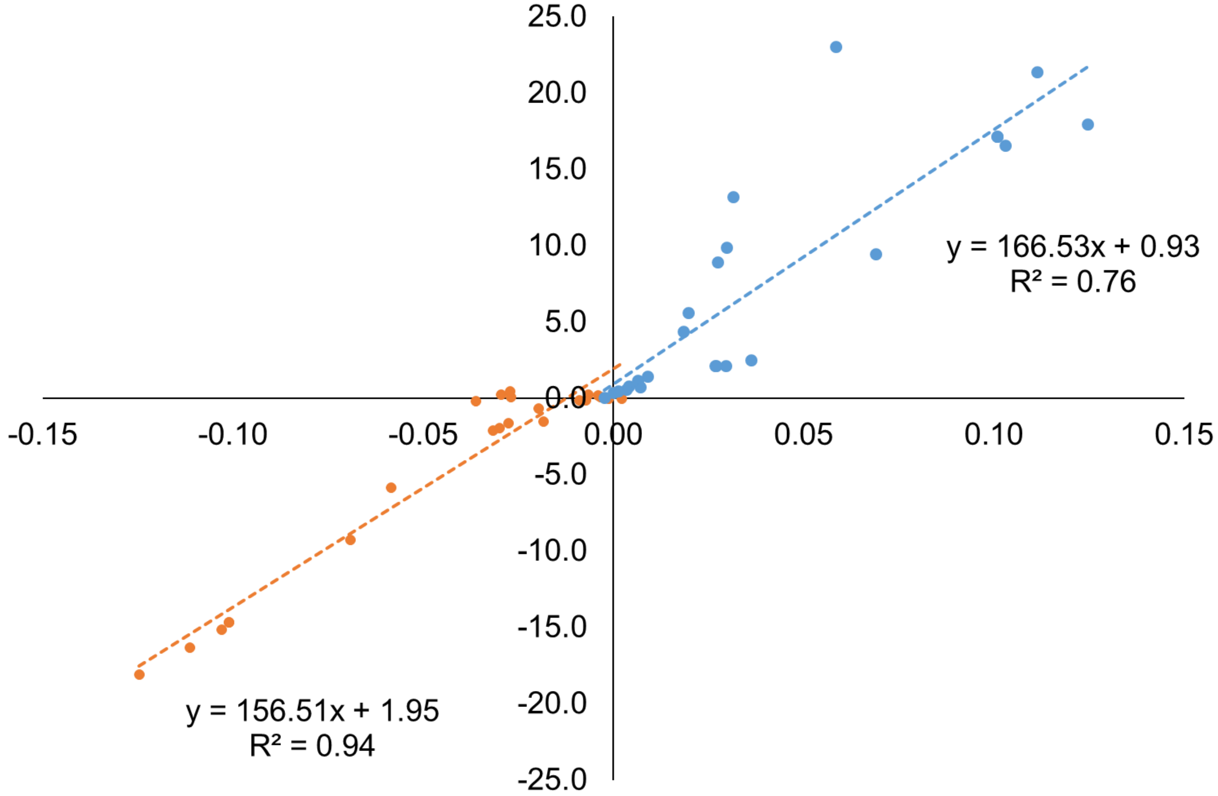

Figure S1. Correlation of $\Delta\varepsilon_{orb,A}$ (in orange) and $\Delta\varepsilon_{orb,B}$ (in blue) (y-axis, in kcal/mol) with the corresponding fragment charge difference between the *AB* and A^0B^0 states (x-axis, in electrons).

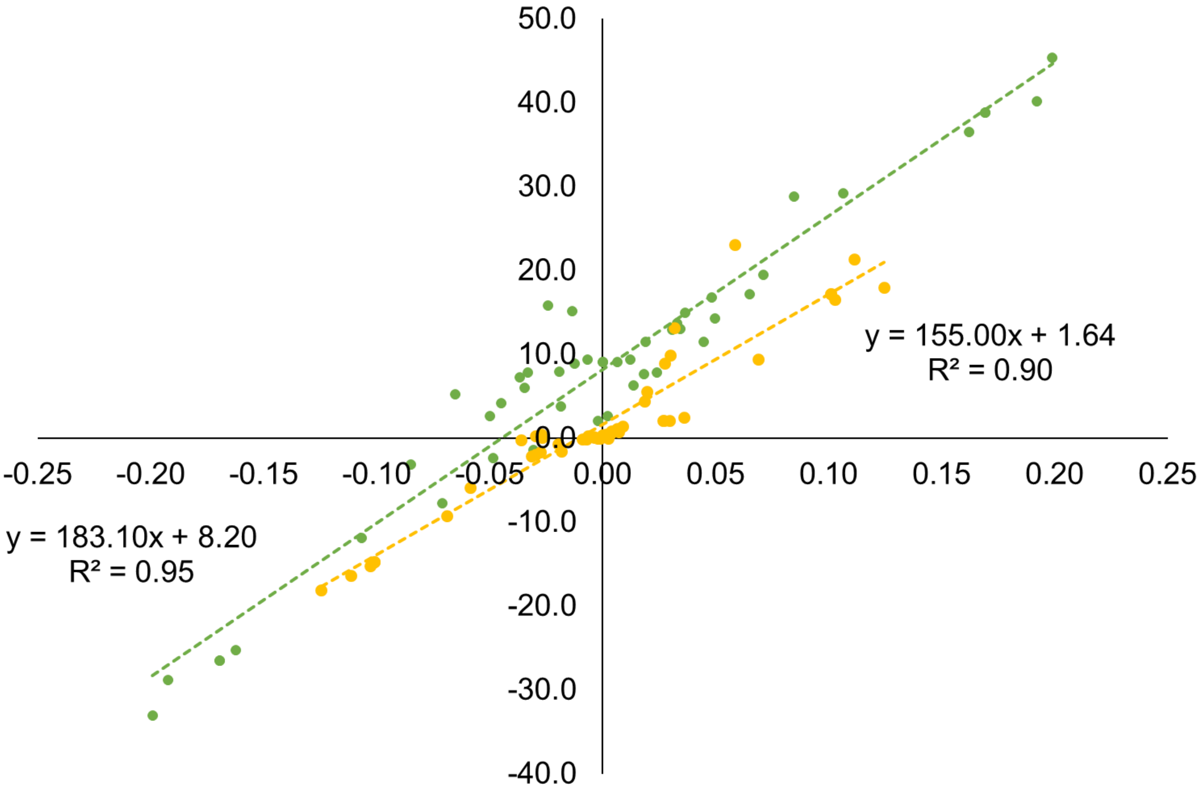

Figure S2. Correlation (in green) of $\Delta\varepsilon_{\mathrm{def,el,A}}$ and $\Delta\varepsilon_{\mathrm{def,el,B}}$ (y-axis, in kcal/mol) with the corresponding fragment charge difference between the *AB* and A^0 and B^0 states (x-axis, in electrons). Correlation (in yellow) of $\Delta\varepsilon_{\mathrm{orb,A}}$ and $\Delta\varepsilon_{\mathrm{orb,B}}$ (y-axis, in kcal/mol) with the corresponding fragment charge difference between the *AB* and A^0B^0 states (x-axis, in electrons).

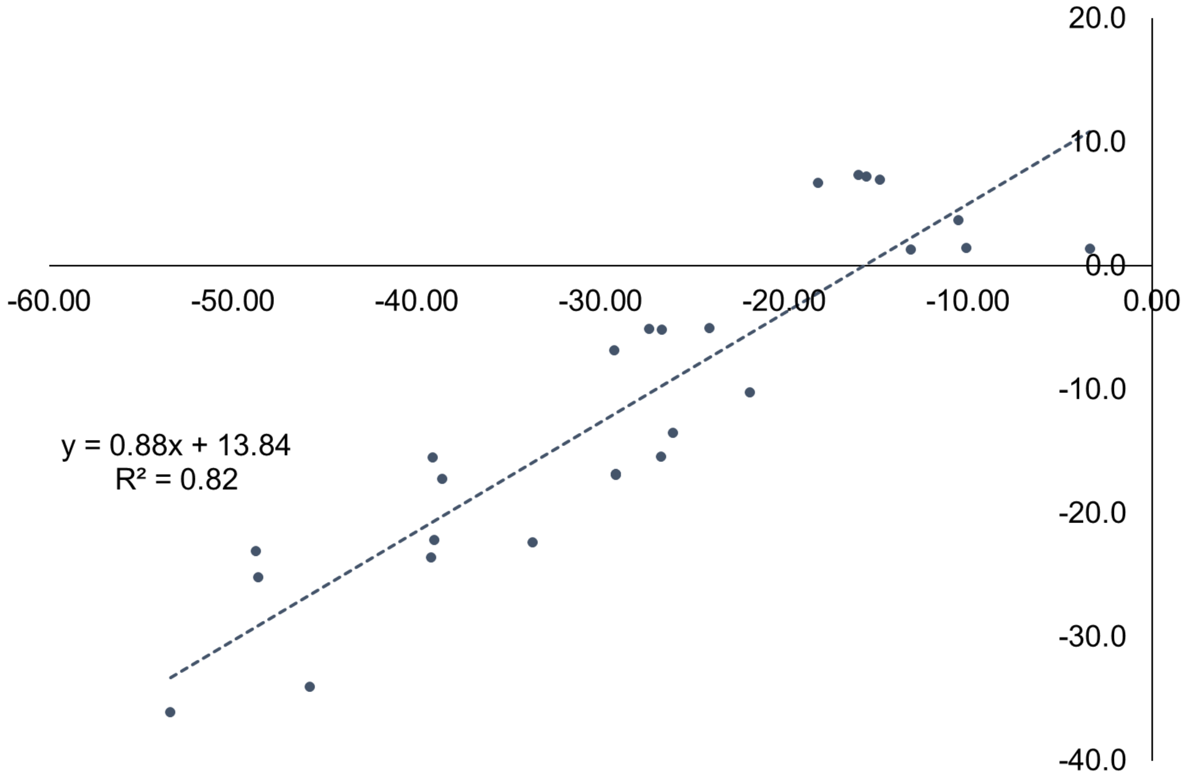

Figure S3. Correlation (in grey) of ΔE_{int} (y-axis, in kcal/mol) with $\Delta \varepsilon_{\text{int,AB}}$ (x-axis, in kcal/mol).

Table S1. Fragment charges (TFVC) for each of the states of the EDA process. DMA = dimethylacetamide.

		AB		*A⁰B⁰*		*A⁰*	*B⁰*
A=Acceptor	B=Donor	A	B	A	B	A	B
H_2O	H_2O	-0.035	0.035	-0.008	0.008	0.000	0.000
H_2O	MeOH	-0.033	0.033	-0.006	0.006	0.000	0.000
MeOH	MeOH	-0.037	0.037	-0.007	0.007	0.000	0.000
H_2O	NH_3	-0.065	0.065	-0.029	0.029	0.000	0.000
NH_4^+	H_2O	0.915	0.085	0.984	0.016	1.000	0.000
Li^+	H_2O	0.929	0.071	0.956	0.044	1.000	0.000
Na^+	H_2O	0.955	0.045	0.975	0.025	1.000	0.000
K^+	H_2O	0.986	0.014	1.005	-0.005	1.000	0.000
NH_4^+	C_4H_4S	0.801	0.199	0.912	0.088	1.000	0.000
NH_4^+	C_6H_6	0.831	0.169	0.932	0.068	1.000	0.000
NH_4^+	C_4H_4O	0.838	0.162	0.941	0.059	1.000	0.000
NH_4^+	C_4H_4NH	0.808	0.192	0.932	0.068	1.000	0.000
NH_4^+	C_6H_6	0.831	0.169	0.932	0.068	1.000	0.000
Li^+	C_6H_6	0.893	0.107	0.952	0.048	1.000	0.000
Na^+	C_6H_6	0.950	0.050	0.982	0.018	1.000	0.000
K^+	C_6H_6	0.976	0.024	1.006	-0.006	1.000	0.000
C_6H_6	C_6H_6	0.000	0.000	0.000	0.000	0.000	0.000
C_5H_5N	C_6H_6	-0.007	0.007	-0.003	0.003	0.000	0.000
$C_4H_4N_2$	C_6H_6	-0.012	0.012	-0.003	0.003	0.000	0.000
DMA	C_6H_6	-0.019	0.019	-0.012	0.012	0.000	0.000
C_6H_6	C_6H_6 (T)	-0.018	0.018	-0.011	0.011	0.000	0.000
C_6H_6	C_6H_5F	-0.002	0.002	-0.004	0.004	0.000	0.000
C_6H_6	C_6H_5Cl	0.031	-0.031	0.032	-0.032	0.000	0.000
C_6H_6	C_6H_5Br	0.048	-0.048	0.052	-0.052	0.000	0.000
C_6F_6	F^-	-0.135	-0.865	-0.024	-0.976	0.000	-1.000
C_6F_6	Cl^-	-0.176	-0.824	-0.090	-0.910	0.000	-1.000
C_6F_6	Br^-	-0.201	-0.799	-0.132	-0.868	0.000	-1.000

Table S2. Fragment charge differences (TFVC) associated to each energy term of the EDA process. DMA = dimethylacetamide.

		Pauli		Orb. Int.		Int.	
A=Acceptor	B=Donor	A	B	A	B	A	B
H_2O	H_2O	-0.008	0.008	-0.027	0.027	-0.035	0.035
H_2O	MeOH	-0.006	0.006	-0.027	0.027	-0.033	0.033
MeOH	MeOH	-0.007	0.007	-0.030	0.030	-0.037	0.037
H_2O	NH_3	-0.029	0.029	-0.036	0.036	-0.065	0.065
NH_4^+	H_2O	-0.016	0.016	-0.069	0.069	-0.085	0.085
Li^+	H_2O	-0.044	0.044	-0.027	0.027	-0.071	0.071
Na^+	H_2O	-0.025	0.025	-0.020	0.020	-0.045	0.045
K^+	H_2O	0.005	-0.005	-0.018	0.018	-0.014	0.014
NH_4^+	C_4H_4S	-0.088	0.088	-0.111	0.111	-0.199	0.199
NH_4^+	C_6H_6	-0.068	0.068	-0.101	0.101	-0.169	0.169
NH_4^+	C_4H_4O	-0.059	0.059	-0.103	0.103	-0.162	0.162
NH_4^+	C_4H_4NH	-0.068	0.068	-0.125	0.125	-0.192	0.192
NH_4^+	C_6H_6	-0.068	0.068	-0.101	0.101	-0.169	0.169
Li^+	C_6H_6	-0.048	0.048	-0.058	0.058	-0.107	0.107
Na^+	C_6H_6	-0.018	0.018	-0.032	0.032	-0.050	0.050
K^+	C_6H_6	0.006	-0.006	-0.030	0.030	-0.024	0.024
C_6H_6	C_6H_6	0.000	0.000	0.000	0.000	0.000	0.000
C_5H_5N	C_6H_6	-0.003	0.003	-0.004	0.004	-0.007	0.007
$C_4H_4N_2$	C_6H_6	-0.003	0.003	-0.009	0.009	-0.012	0.012
DMA	C_6H_6	-0.012	0.012	-0.006	0.006	-0.019	0.019
C_6H_6	C_6H_6 (T)	-0.011	0.011	-0.007	0.007	-0.018	0.018
C_6H_6	C_6H_5F	-0.004	0.004	0.002	-0.002	-0.002	0.002
C_6H_6	C_6H_5Cl	0.032	-0.032	-0.001	0.001	0.031	-0.031
C_6H_6	C_6H_5Br	0.052	-0.052	-0.003	0.003	0.048	-0.048
C_6F_6	F^-	-0.024	0.024	-0.111	0.111	-0.135	0.135
C_6F_6	Cl^-	-0.090	0.090	-0.086	0.086	-0.176	0.176
C_6F_6	Br^-	-0.132	0.132	-0.069	0.069	-0.201	0.201

Table S3. Fragment (IQA) decomposition of each energy term from EDA for some selected systems using the TFVC and Hirshfeld-Iterative atom in molecule definitions. All the energies are given in kcal/mol.

		Elstat.			Pauli			Orb. Int.			Int.		
A	B	A	B	AB	A	B	AB	A	B	AB	A	B	AB
TFVC													
H_2O	H_2O	-6.1	-0.6	-2.2	12.0	11.5	-15.5	0.1	2.1	-6.4	6.0	13.1	-24.1
H_2O	MeOH	-6.8	-0.9	-1.6	14.2	12.5	-17.2	0.5	2.1	-7.8	7.8	13.7	-26.7
Li^+	H_2O	-26.7	0.1	-7.3	20.6	10.5	-18.4	-1.6	8.9	-20.0	-7.7	19.5	-45.8
Na^+	H_2O	-17.5	0.2	-8.1	22.4	5.6	-19.7	-0.7	5.6	-11.4	4.2	11.5	-39.2
Hirshfeld-I													
H_2O	H_2O	-7.8	-1.0	-0.1	17.4	21.6	-30.8	-1.3	2.4	-5.3	8.3	23.0	-36.2
H_2O	MeOH	-9.1	-1.7	1.3	18.3	24.2	-33.0	-1.1	2.3	-6.4	8.1	24.9	-38.0
Li^+	H_2O	-15.4	0.9	-19.5	72.1	-18.2	-41.3	4.5	8.8	-26.3	61.2	-8.4	-87.0
Na^+	H_2O	-12.3	1.0	-14.1	55.8	-10.4	-37.1	2.8	5.2	-14.3	46.4	-4.1	-65.6
